# Hybrid Solid Oxide Cell Enables Efficient and Robust High-Temperature CO_2_ Electrolysis

**DOI:** 10.1021/jacs.6c15391

**Published:** 2026-10-23

**Authors:** Wenchao Ma, Jiaming Tian, Jiyuan Wang, Xile Hu

**Affiliations:** † State Key Laboratory of Coordination Chemistry, Key Laboratory of Mesoscopic Chemistry of Ministry of Education, School of Chemistry and Chemical Engineering, 12581 Nanjing University, Nanjing 210023, China; ‡ Laboratory of Inorganic Synthesis and Catalysis, Institute of Chemical Sciences and Engineering, École Polytechnique Fédérale de Lausanne (EPFL), Lausanne 1015, Switzerland

## Abstract

Electrocatalytic conversion of CO_2_ into chemicals and fuels offers a promising route toward a circular carbon economy. High-temperature CO_2_ electroreduction in solid oxide electrolysis cells (SOECs) delivers high selectivity and energy efficiency, but balancing mechanical robustness and electrochemical performance remains challenging. Here, we address this challenge by introducing a hybrid ionic-conductor architecture that spatially decouples electrocatalysis, ionic transport, and mechanical support into function-specific layers. The resulting cell combines high mechanical robustness (fracture load > 7 N) with 1.5–3.0-fold higher CO_2_-to-CO activity than state-of-the-art SOECs over 600–800 °C. Stable operation is maintained for over 1000 h at 1.0 A cm^–2^. Furthermore, we demonstrate scalability with a single-cell area of 100 cm^2^, achieving total currents up to 120 A and powers up to 200 W. This work establishes an effective strategy for developing mechanically resilient, efficient, and scalable energy conversion devices.

## Graphical abstract

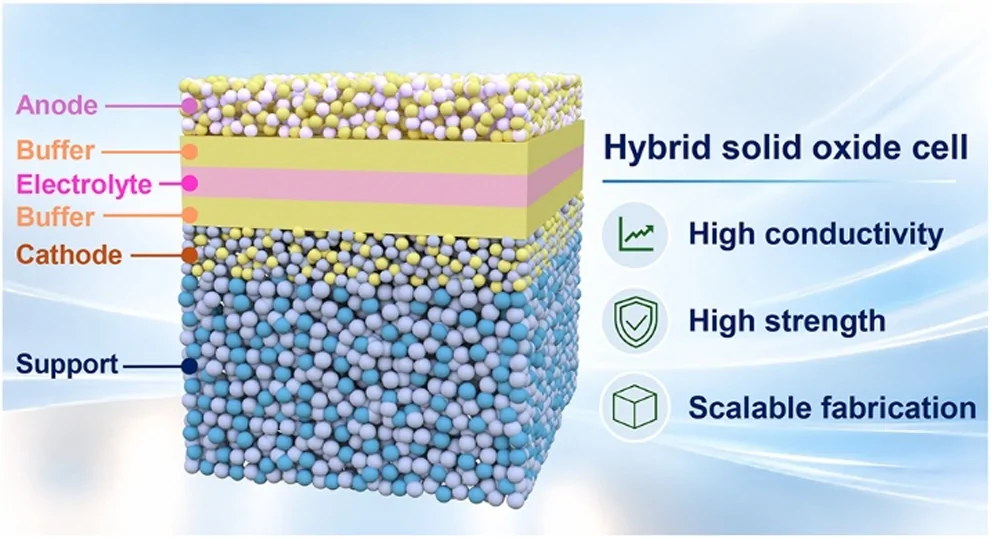

## Introduction

Electrocatalytic conversion of CO_2_ into value-added chemicals and fuels holds great promise for carbon utilization and renewable energy storage.^1,2^ Among available technologies, high-temperature electrolysis (600–1000 °C) in solid oxide electrolysis cells (SOECs) stands out for its exceptional product selectivity, energy efficiency and operational stability at industrially relevant current densities.^3–5^ Although SOEC configurations for CO_2_ and H_2_O electrolysis are similar, their performance differs markedly.^5–7^ H_2_O electrolysis has achieved substantial progress, with energy efficiencies exceeding 90% and lifetimes beyond 10,000 h at current densities of ≥1.0 A cm^–2^.^8,9^ In contrast, CO_2_ electrolysis remains significantly more challenging, typically delivering ∼70% efficiency and ∼1000 h stability under comparable conditions.^10,11^ This disparity arises from intrinsically slower reaction kinetics, more limited gas transport and susceptibility to coking in CO_2_ electrolysis.^5–10^ Moreover, high performance in CO_2_ SOEC is often achieved at the expense of mechanical robustness, as the use of thin support layers or highly conductive materials compromises structural strength.^6,10,11^ Ensuring sufficient mechanical integrity while maintaining high electrochemical activity remains a key challenge for the practical deployment of SOEC technology.

Current SOECs for CO_2_ electrolysis can be categorized into electrolyte-supported cells (ESCs) and cathode-supported cells (CSCs).^6,12^ ESCs are simple to fabricate and offer good electrode–electrolyte material compatibility, but their thick electrolyte layers (80–500 μm) lead to high ohmic resistance and low electrolysis activity (Figure 1). In contrast, CSCs employ thin electrolytes (typically ≤20 μm) to reduce resistance, but the use of dissimilar oxygen-ion conductors in the support, cathode, and electrolyte layers often results in poor compatibility during cosintering. This constraint typically necessitates the use of identical oxygen-ion conductors in all three layers, forming monolithic-structured cathode-supported cells (MCSCs) to prevent shrinkage mismatch during fabrication^13–15^ (Figure 1). Several strategies, including catalyst design,^11,16–19^ electrolyte modification,^20,21^ thickness engineering,^22,23^ and geometry and assembly optimization,^2,24^ have been explored to enhance performance. Nevertheless, both ESCs and CSCs continue to face a trade-off between mechanical strength and electrochemical activity. For a 1 × 2 cm^2^ cell with a fracture load exceeding 5 N, the achievable current density for CO_2_ electrolysis typically remains below 1.0 A cm^–2^ at an industrial-relevant voltage of 1.3 V (Table S1).

**Figure 1:**
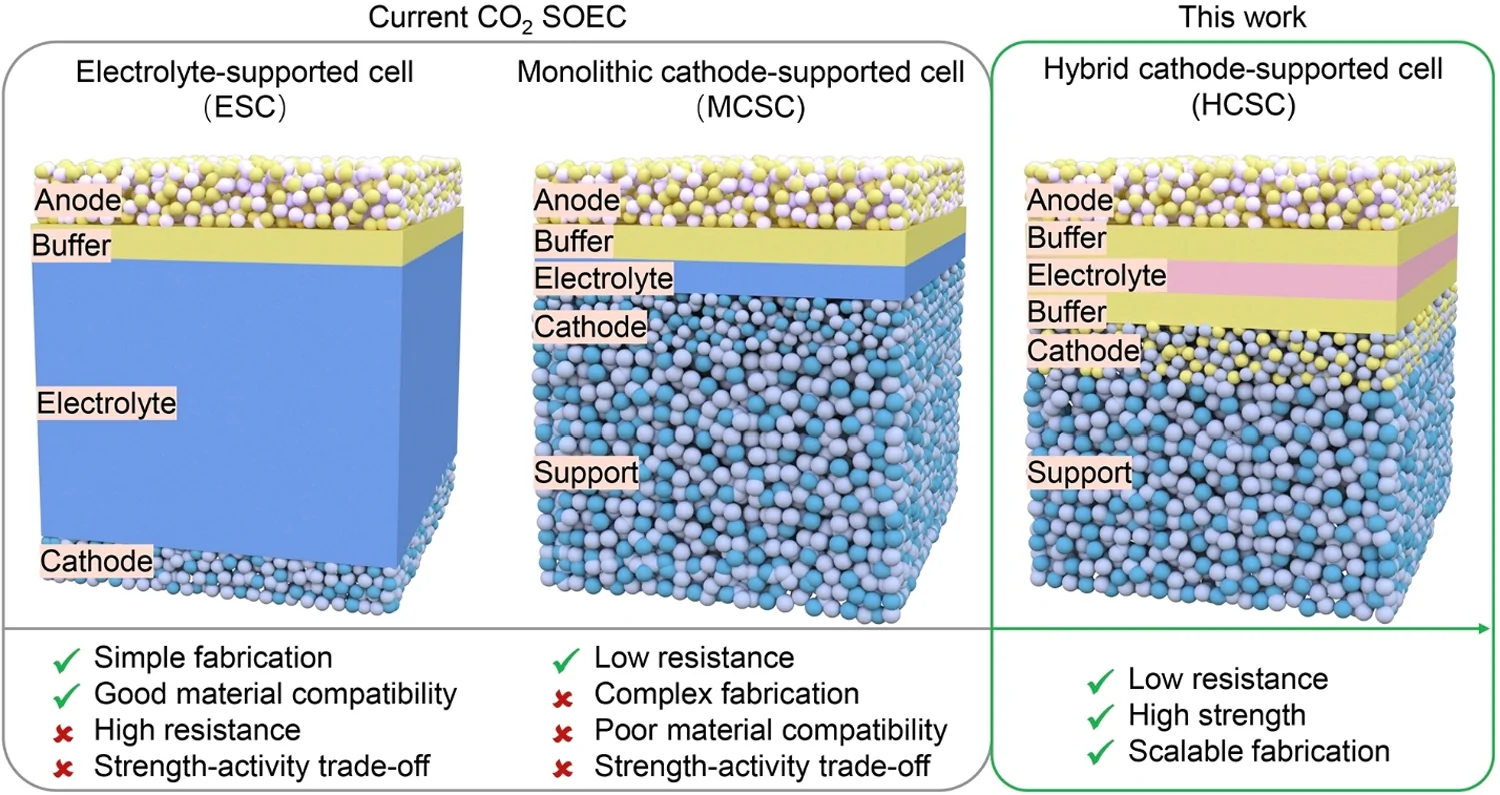
Architectural designs of SOECs for CO_2_ electrolysis. Comparison between the cell architectures developed in this work and representative state-of-the-art SOEC designs. Gray balls represent Ni. All other colored balls or plates represent oxide phases, with each color corresponding to a distinct oxide.

Here we propose a hybrid-structured, cathode-supported cell (HCSC) that establishes a functionally decoupled architecture by spatially integrating distinct oxygen-ion conductors across different layers (Figure 1). Rather than relying on the discovery of new materials, this design leverages commercially available oxides to independently optimize mechanical strength, ionic conductivity, and electrocatalytic activity. Specifically, a high-strength conductor, ScSZ ((Sc_2_O_3_)_0.06_(ZrO_2_)_0.94_), serves as the structural support; a mixed ionic-electronic conductor, SDC (Sm_0.2_Ce_0.8_O_2‑δ_), is deployed in the cathode and buffer layers; and a high ionic conductor, ScCeSZ ((Sc_2_O_3_)_0.10_(CeO_2_)_0.01_(ZrO_2_)_0.89_), functions as the electrolyte. This combination yields a fracture load of 7.3 N for a 1 × 2 cm^2^ area, while delivers 1.5–3.0-fold higher CO_2_-to-CO activity than state-of-the-art SOECs over operating temperatures of 600–800 °C. Furthermore, we demonstrate scalability with a single-cell area of 100 cm^2^, achieving a total current of 120 A and power of 200 W. This hybrid structural design reconciles mechanical robustness with catalytic efficiency, advancing SOEC technology toward practical industrial implementation.

## Results and Discussion

### Design of a Hybrid-Structured Cell

A typical cathode-supported SOEC comprises several functional layers: a metal-oxide support providing mechanical integrity, a metal-oxide cathode for CO_2_ reduction to CO and O^2–^, an oxide electrolyte for oxygen-ion transport, an oxide buffer layer to prevent interfacial reactions between the electrolyte and anode, and an oxide anode for O^2–^ oxidation to O_2_ (Figure S1).^13,14^ Nickel oxide is the most widely used precursor to the metal component in the support and cathode because it can be in situ reduced to nickel, which combines high catalytic efficiency for high-temperature CO_2_ electroreduction with good chemical compatibility with various oxides.^3,6^ The oxide phases serve as oxygen-ion conductors, each tailored to perform distinct electrochemical or structural roles, and thus represent the functional core of the cell.^12,22^ During fabrication, the support, cathode, electrolyte, and buffer layers are typically cosintered at high temperatures to densify the electrolyte and minimize interfacial resistance, while the anode is sintered separately at a lower temperature to avoid thermal degradation.^13,14^ Consequently, shrinkage mismatch between the anode and other layers is generally negligible.

To understand why the MCSCs, which employ identical oxides in the support, cathode and electrolyte layers, exhibit a trade-off between mechanical strength and electrochemical activity, we investigated six commercially available oxygen-ion conductors: 3YSZ ((Y_2_O_3_)_0.03_(ZrO_2_)_0.97_), 8YSZ ((Y_2_O_3_)_0.08_(ZrO_2_)_0.92_), ScSZ ((Sc_2_O_3_)_0.06_(ZrO_2_)_0.94_), LSGM (La_0.8_Sr_0.2_Ga_0.8_Mg_0.2_O_3‑δ_), ScCeSZ ((Sc_2_O_3_)_0.10_(CeO_2_)_0.01_(ZrO_2_)_0.89_), and SDC (Sm_0.2_Ce_0.8_O_2‑δ_). The measured flexural strengths followed the order SDC < ScCeSZ < LSGM < 8YSZ ≪ 3YSZ < ScSZ (Figure 2A), whereas their ionic conductivities increased in sequence as 3YSZ < 8YSZ < ScSZ < LSGM < ScCeSZ < SDC (Figure 2B), consistent with the trend reported in the literature.^25,26^ This correlation demonstrates that higher flexural strength is invariably accompanied by lower ionic conductivity, thereby explaining the intrinsic mechanical strength-activity trade-off observed in MCSCs. NiO, although highly conductive electronically after reduction to Ni, possesses low flexural strength and thus cannot provide sufficient mechanical support (Figure S2). Previous attempts to combine different oxygen-ion conductors within cathode-supported architectures yielded only modest gains, likely due to suboptimal material combinations and processing incompatibilities.^20,27,28^

**Figure 2:**
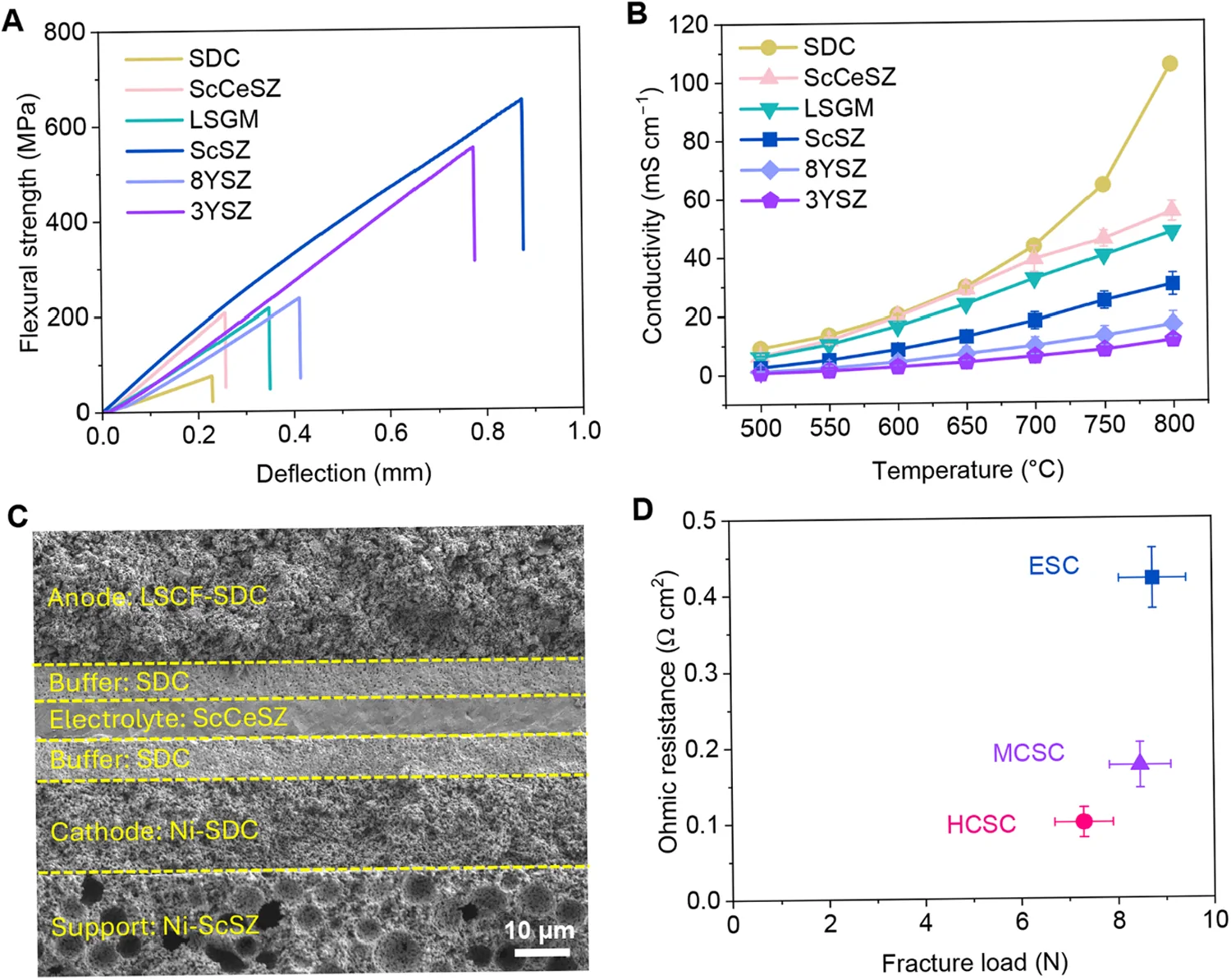
A hybrid-structured SOEC design. (A) Flexural strength of different oxygen-ion conductors. (B) Ionic conductivity of different oxygen-ion conductors. (C) Cross-sectional SEM image of the HCSC. (D) Comparison of ohmic resistance at 800 °C and fracture load among different cells. The results are shown as the mean ± s.d. from three individual experiments.

Building on these trends, we designed a hybrid-structured solid oxide cell that strategically integrates different oxygen-ion conductors to decouple mechanical strength from electrocatalysis and ionic conductivity (Figure S3). ScSZ, possessing the highest flexural strength, was selected as the mechanical support material. SDC, which exhibits the highest ionic conductivity but also partial electronic conduction at high temperatures,^29^ was employed in the cathode and buffer layers where mixed conduction is beneficial but excluded from the electrolyte. Instead, ScCeSZ, offering the second-highest ionic conductivity with negligible electronic leakage,^30^ was adopted as the electrolyte material to ensure efficient oxygen-ion transport.

This combination had not been previously realized, likely due to the shrinkage mismatch among different oxygen-ion conductors.^25,31^ The measured linear shrinkage followed the order SDC > ScSZ > ScCeSZ (Figure S4). As expected, a simple assembly with the structure **Ni-ScSZ** | Ni-SDC | ScCeSZ | SDC (**support** | cathode | electrolyte | buffer; font for support is bold) exhibited severe warping toward the buffer layer and delamination between the electrolyte and buffer (Figure S3A). By sandwiching the Ni-ScSZ support between two Ni-SDC layers (**Ni-SDC** | **Ni-ScSZ** | Ni-SDC | ScCeSZ | SDC), warping was effectively eliminated (Figure S3B). Further sandwiching the ScCeSZ electrolyte between two SDC layers (**Ni-SDC** | **Ni-ScSZ** | Ni-SDC | SDC | ScCeSZ | SDC) mitigated delamination (Figure S3C). The support **Ni-SDC** layer was subsequently polished, and the thin SDC buffer layer between the cathode and electrolyte was retained, as its high ionic conductivity minimally affects cell performance. It should be noted that the particle size, surface area, and green packing density of the starting powders can also influence sintering shrinkage. Further optimization of these powder characteristics and processing conditions may therefore reduce the shrinkage mismatch among the constituent layers and further improve the fabrication quality and performance of the HCSC.

Finally, a composite of La_0.6_Sr_0.4_Co_0.2_Fe_0.8_O_3‑δ_ (LSCF)-SDC anode^32^ was deposited to complete the HCSC, yielding the final structure: **Ni-ScSZ** | Ni-SDC | SDC | ScCeSZ | SDC | LSCF-SDC (Figure S5 and Table S2). For comparison, two reference cells were also fabricated: a monolithic-structured cathode-supported cell (MCSC, **Ni-ScSZ** | Ni-ScSZ | ScSZ | SDC | LSCF-SDC; Figure S6) and an electrolyte-supported cell ESC, Ni-ScSZ | **ScSZ** | SDC | LSCF-SDC (Figure S7).

Cross-sectional scanning electron microscopy (SEM) images revealed the dense electrolyte and buffer layers and no interfacial delamination in any of the three architectures (Figures 2C and S8). Open circuit voltage values of ∼1.14 V under H_2_–O_2_ fuel-cell conditions further confirm that the electrolytes are sufficiently gastight for electrochemical operation (Figure S9). The support layers in both HCSC and MCSC were porous to facilitate gas transport, while their cathode layers were comparatively compact, providing abundant active sites (Figures 2C and S8). Mechanical testing showed comparable fracture loads of 7.3–8.8 N for 1 × 2 cm^2^ cells (Figures 2D and S10A), indicating similarly high mechanical strength across all architectures. In contrast, the measured ohmic resistances at 800 °C were 0.10, 0.17, 0.42 Ω cm^2^ for the HCSC, MCSC, and ESC, respectively (Figures 2D and S10B). These results demonstrate that the HCSC achieves the lowest resistance under comparable mechanical strength.

### High-Temperature Electrochemical Measurements

To evaluate the electrochemical performance of different cell configurations, CO_2_ electrolysis to CO was carried out over a temperature range of 600–800 °C. The hybrid-structured cell (HCSC) exhibited the highest activity among all tested configurations, even after ohmic resistance correction (Figures 3A and S11). At a cell voltage of 1.3 V, the HCSC achieved current densities of 2.5, 1.1, and 0.36 A cm^–2^ at 800, 700, and 600 °C, respectively. Under identical conditions, the monolithic-structured cell (MCSC) delivered 1.7, 0.71, and 0.24 A cm^–2^, whereas the electrolyte-supported cell (ESC) reached only 0.67, 0.37, and 0.15 A cm^–2^. Accordingly, the HCSC outperformed the MCSC by factors of 1.3–1.6 and the ESC by factors of 1.8–3.7, depending on the temperature. Notably, the HCSC also exhibited superior H_2_–O_2_ fuel cell performance compared with the MCSC (Figure S9).

**Figure 3:**
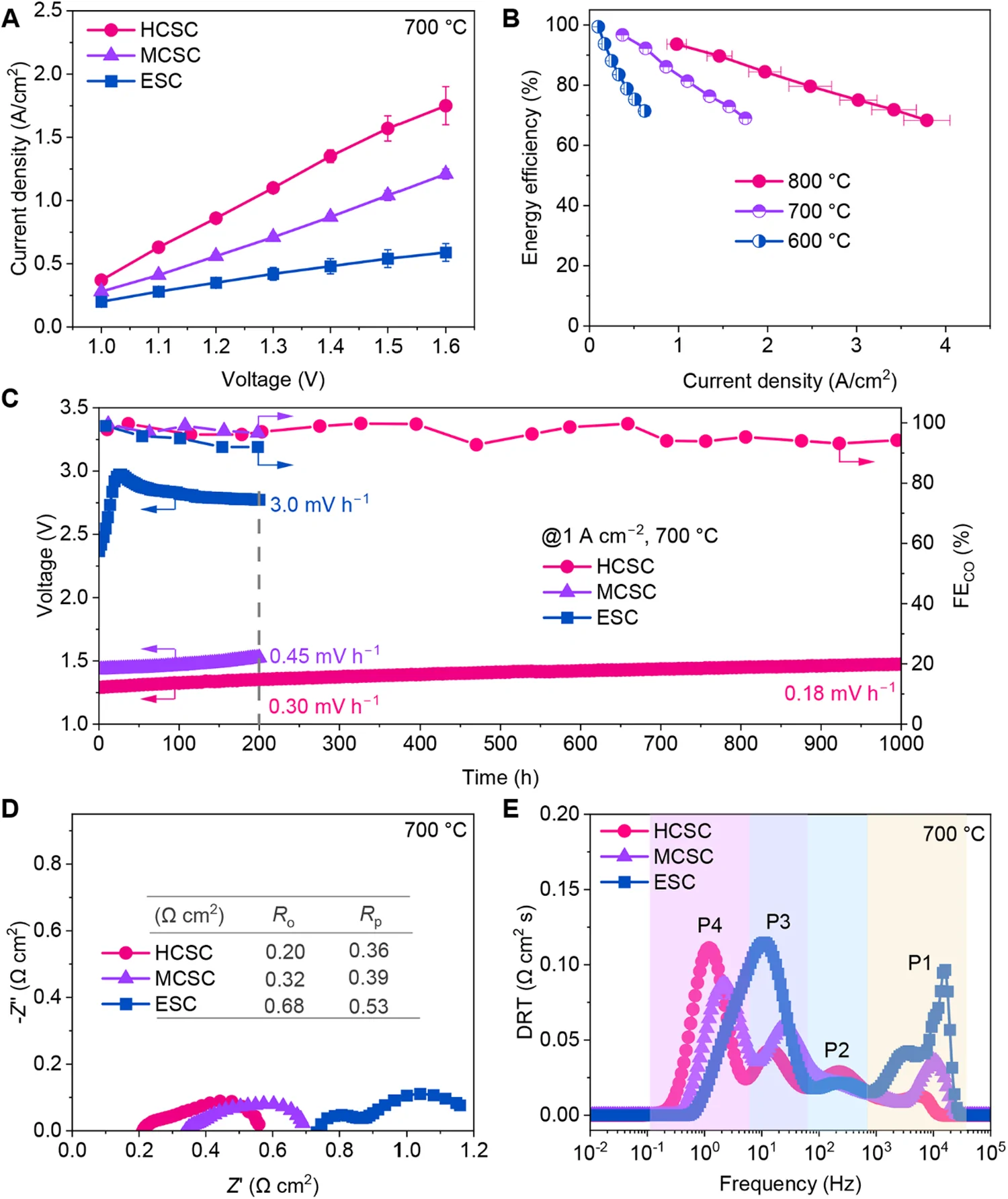
High-temperature electrochemical measurements. (A) Current density–voltage curves of different cells at 700 °C. (B) Energy efficiency as a function of current density for different cells at different temperatures. (C) Stability tests at a constant current density of 1.0 A cm^–2^ at 700 °C. (D) EIS spectra and fitted results for different cells at 1.0 V and 700 °C. (E) DRT analyses for different cells at 1.0 V and 700 °C. The results are shown as the mean ± s.d. from three individual experiments.

The energy efficiency exhibited the same trend of HCSC > MCSC > ESC across all examined temperatures (Figures 3B and S11). The HCSC maintained ≥80% energy efficiency at current densities up to 2.5 A cm^–2^ at 800 °C. Although efficiency gradually decreased at higher current densities due to an increased cell voltage, values remained ≥68% even at 3.8 A cm^–2^, demonstrating robust electrochemical performance under high current densities. Lowering the operating temperature produced slightly higher energy efficiencies at a given voltage but reduced current densities, consistent with thermodynamic expectations.^3^

Operational stability is another metric for assessing the viability of high-rate CO_2_ electrolysis systems. Long-term tests (Figure 3C) were conducted at an industrially relevant current density of 1.0 A cm^–2^ and 700 °C, a temperature selected for its favorable interconnector stability compared with operation at 800 °C.^33^ Over 200 h of operation, the HCSC exhibited the smallest voltage increase, from 1.29 to1.35 V, corresponding to a low degradation rate of 0.30 mV h^–1^. The MCSC showed a slightly larger increase, from 1.43 to1.52 V, yielding a degradation rate of 0.45 mV h^–1^. In contrast, the ESC degraded rapidly, with its voltage increasing from 2.4 to 3.0 V within the first 30 h and remaining near 2.8 V thereafter, giving an average degradation rate of 3.0 mV h^–1^. All three cells maintained nearly 100% Faradaic efficiencies for CO (FE_CO_) throughout the test. Extending the HCSC operation to 1000 h resulted in only a modest voltage increase to 1.47 V, corresponding to a low long-term degradation rate of 0.18 mV h^–1^. In addition, the HCSC exhibited greater tolerance to pressure loading under operating conditions than the conventional LSGM-based ESC (Figure S12). These results highlight the superior durability of the hybrid-structured cell over other configurations under high-temperature CO_2_ electrolysis.

To elucidate the origin of performance differences among the three architectures, operando electrochemical impedance spectroscopy (EIS) and distribution of relaxation time (DRT) analyses were performed. The EIS results (Figures 3D and S13) showed that both the ohmic resistances (*R*_0_) and polarization resistances (*R*_p_) followed the order HCSC < MCSC < ESC, indicating progressively greater ohmic and polarization losses from hybrid to monolithic to electrolyte-supported designs. Three cells exhibited similar double-layer capacitances (Figure S13). The DRT spectra (Figure 3E) resolved four relaxation processes, which were labeled P1–P4 from high to low frequency. Controlled experiments showed that increasing the cathode CO_2_ concentration predominantly decreased the intensity of P4 and also affected P3, whereas increasing the anode O_2_ concentration increased the intensity of P2 (Figure S14). Increasing the operating temperature decreased the intensities of all four peaks (Figure S14). Based on these controlled experiments and previous reports,^17,34^ P1 was assigned to high-frequency oxygen-ion transport involving the electrolyte and buffer layers, P2 to the oxygen evolution reaction at the anode, P3 to the CO_2_ reduction reaction at the cathode, and P4 to gas transport. The intensity of P1 increased in the order HCSC < MCSC < ESC, consistent with lower *R*_0_ in the hybrid cell. P2 remained nearly identical across all samples, as expected from the same LSCF-SDC anode. The P3 feature, associated with CO_2_ reduction, was notably smaller in the HCSC than in the MCSC and ESC, reflecting faster cathode kinetics with the Ni-SDC catalyst due to its higher ionic conductivity compared with Ni-ScSZ. This is further supported by the lower polarization activation energy^35^ of the HCSC compared to the MCSC (Figure S15). Although MCSC and ESC share the same Ni-ScSZ cathode, the cosintered MCSC likely promotes stronger cathode-electrolyte bonding and improved interfacial transport, resulting in a lower *R*_p_. The more pronounced P4 intensity in the cathode-supported cells arises from longer gas transport pathways relative to the ESC. Overall, these analyses confirm that the superior CO_2_ electrolysis performance of the hybrid-structured cell originates from its reduced ohmic resistance and accelerated cathode reaction kinetics compared to the monolithic and electrolyte-supported designs.

Poststability EIS measurements (Figure S13) showed little increases in *R*_0_ for the HCSC and MCSC (<5%), but a substantial rise for the ESC (76%). All three cells exhibited notable increases in *R*_p_ (>45%). Corresponding DRT spectra (Figure S16) revealed moderate increases in P1 and P2 intensities for both HCSC and MCSC, accompanied by pronounced growth in P3 and P4, indicating degradation primarily rooted in the cathode. The ESC, however, showed substantial increases in P1, P3, and P4 intensities, pointing to additional ionic transport losses not observed in the cathode-supported cells. This enhanced ionic stability in the HCSC and MCSC likely results from the stronger cathode-electrolyte bonding established during cosintering.

Previous studies have reported the susceptibility of Ni to sintering and coke formation during high-temperature CO_2_ electrolysis.^11,27,36^ Energy-dispersive X-ray spectroscopy (EDS) mappings using SEM revealed pronounced Ni particle coarsening in the cathodes of all three cells after long-term operation (Figures S17–S19), supporting Ni coarsening as an important contributor to the observed cathode degradation. Raman measurements further revealed coke formation on the Ni-SDC cathode of the HCSC after long-term operation (Figure S20). No obvious interfacial delamination was observed in any of the three cell architectures (Figure S21), and SEM-EDS elemental mapping revealed no obvious interdiffusion of Sc, Zr, Ce, Sm, or Ni across the interfaces either before or after stability testing (Figure S22). In addition, X-ray diffraction (XRD) measurements showed that the ScSZ support retained its cubic phase without detectable phase transformation after sintering, reduction, and long-term stability testing (Figure S23). Together with the EIS and DRT analyses, these results indicate that cathode degradation, including Ni coarsening and coke formation, is a major contributor to the long-term performance decay, while the ESC additionally exhibits ionic-transport-related degradation. These findings suggest that further improvements in cathode catalyst stability could yield additional gains in long-term durability, though such optimization lies beyond the scope of the present work.

### Scale-Up Demonstration

Scalability is essential for industrial deployment yet remains challenging due to the intrinsic brittleness of SOEC materials.^5^ In our system, the support, electrolyte, and buffer-layer materials are commercially available, and both cathode and anode compositions can be synthesized through simple routes. Moreover, the fabrication steps, including tape casting, lamination and screen printing, are all fairly established.^37^ Together with the enhanced mechanical robustness of the HCSC, these factors enable successful scaling of the single-cell area from 1 to 100 cm^2^ (Figure 4A). The 100 cm^2^ HCSC achieved a total current up to 120 A and a total power up to 200 W for CO_2_-to-CO conversion at 700 °C (Figure 4B). While a 1 cm^2^ cell sectioned from the 100 cm^2^ cell exhibited a current density comparable to that of a standalone 1 cm^2^ cell across the tested voltage range, the full 100 cm^2^ cell showed a 36 ± 2% reduction in current density relative to the smaller cell (Figure S24). EIS spectra and DRT analyses revealed a notable increase in the anodic oxygen evolution polarization resistance in the 100 cm^2^ cell, likely arising from higher internal gas pressure and a longer transport pathway that hinder O_2_ desorption and mass transport (Figure S24). Although further optimization of anode-side gas management is needed in the device level, these results underscore the strong scalability of the HCSC architecture.

**Figure 4:**
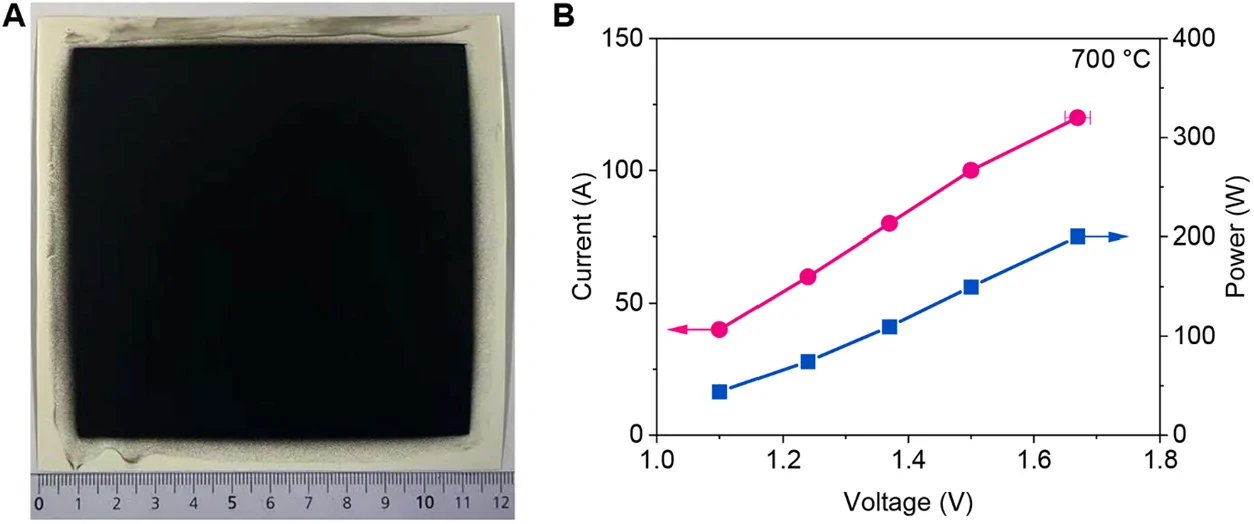
Scale-up demonstration. (A) Photograph of the large HCSC with an active area of 100 cm^2^. (B) Total current and power of the 100 cm^2^ HCSC at 700 °C. The results are shown as the mean ± s.d. from three individual experiments.

### Comparison with Representative SOECs

To benchmark the performance and robustness of our hybrid-structured cell, we compared it with representative SOECs reported for high-temperature CO_2_ electroreduction to CO (Figure 5 and Table S1). As mechanical properties are generally not reported for these cells, their fracture loads were estimated from the support thickness and flexural strengths of the corresponding support materials using Weibull scaling^38^ and are therefore used as a semiquantitative benchmark. The competing requirements of mechanical robustness and electrochemical performance arise from constraints associated with material selection, support thickness, and cell architecture. Achieving industrially relevant current densities (≥1.0 A cm^–2^) typically requires thin and mechanically fragile supports (<200 μm), corresponding to estimated fracture loads typically below ∼5 N for a 1 × 2 cm^2^ cell.^11,13,19^ When support thicknesses are increased to 200–500 μm to improve strength, the current densities generally fall below 1.0 A cm^–2^ at 1.3 V and 800 °C.^14,20,39^ Although thickening the support while maintaining a thin electrolyte might enhance strength without sacrificing ohmic resistance, this approach increases material cost and exacerbates gas-transport resistance.^40–42^ In contrast, our hybrid-structured cell design achieves both robust mechanical integrity and high activity. With a support thickness <300 μm, the HCSC exhibits an experimentally measured fracture load of 7.3 N, placing it in the high-strength regime of the cells benchmarked in Figure 5, while delivering current densities of 2.5, 1.1, and 0.36 A cm^–2^ at 800, 700, and 600 °C, respectively, at 1.3 V. This performance even matches or exceeds that of state-of-the-art CO_2_ SOECs operating at temperatures ∼100 °C higher. In addition, our system demonstrates scalability to a single-cell size of 100 cm^2^. Together, these results highlight the hybrid cell as a promising platform for robust, efficient, and scalable CO_2_ conversion.

**Figure 5:**
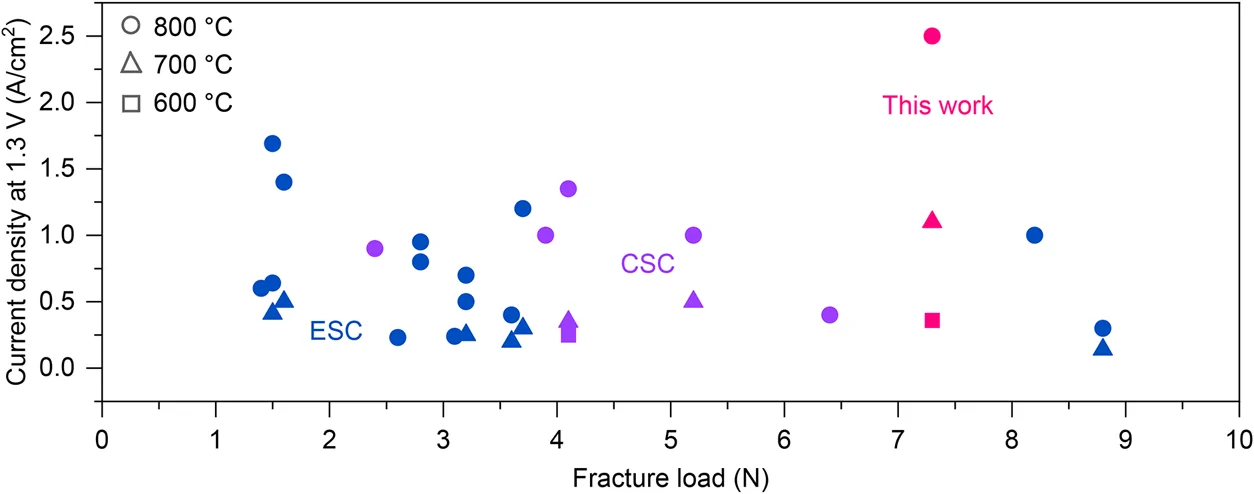
Comparison of electrolysis activity (current density at 1.3 V) and fracture load between the present cells and representative SOECs reported in the literature. Fracture loads of the present cells were experimentally measured, whereas those of the literature-reported cells were estimated from the reported support thicknesses and flexural strength of the corresponding support materials using Weibull scaling, assuming a cell size of 1 × 2 cm^2^. The estimated values are intended to provide a semiquantitative comparison rather than a precise ranking of mechanical strength (see Table S1 for details).

## Conclusions

In summary, we have developed a hybrid-structured, cathode-supported solid oxide cell (**Ni-ScSZ** | Ni-SDC | SDC | ScCeSZ | SDC | LSCF-SDC) for high-temperature CO_2_ electrolysis. By strategically integrating distinct oxygen-ion conductors in different functional layers, this architecture decouples electrocatalysis and ionic transport from mechanical strength, achieving both high structural robustness and superior catalytic performance. The hybrid architecture also enables a 100-fold scale-up in single-cell area. This work provides an effective strategy to unify mechanical strength and catalytic efficiency in solid oxide cells, advancing their potential for practical industrial deployment.

## Supplementary Material



## Notes

Detailed experimental procedures and characterization data (Figures S1–S24, Tables S1 and S2) (PDF)
