# Supplementary material for: Hybrid Solid Oxide Cell Enables Efficient and Robust High-Temperature CO2 Electrolysis
Source: J Am Chem Soc. 2026 Oct 23;148(43):47200–7. doi: 10.1021/jacs.6c15391 (PMC13741827; doi:10.1021/jacs.6c15391)
Supplement: Supplementary file 1 [file ja6c15391_si_001.pdf]

Supporting Information

Hybrid Solid Oxide Cell Enables Efficient and Robust High-Temperature CO_2 Electrolysis

Authors: Wenchao Ma[1,2], Jiaming Tian[2], Jiyuan Wang[1], Xile Hu[2]*

Affiliations:

[1]State Key Laboratory of Coordination Chemistry, Key Laboratory of Mesoscopic Chemistry of Ministry of Education, School of Chemistry and Chemical Engineering, Nanjing University, Nanjing, 210023, China.

[2]Laboratory of Inorganic Synthesis and Catalysis, Institute of Chemical Sciences and Engineering, École Polytechnique Fédérale de Lausanne (EPFL), Lausanne, 1015, Switzerland.

*Corresponding author. Email: xile.hu@epfl.ch

Chemicals and materials

Ammonia solution (25%, VWR), ethylenediaminetetraacetic acid (EDTA, 99%, IVALUA), nickel(II) nitrate hexahydrate ($Ni(NO_3)_2\cdot6H_2O$, 99.9%, ABCR), cerium(III) nitrate hexahydrate ($Ce(NO_3)_3\cdot6H_2O$, 99.5%, ABCR), samarium(III) nitrate hexahydrate ($Sm(NO_3)_3\cdot6H_2O$, 99.9%, ABCR), lanthanum(III) nitrate hexahydrate ($La(NO_3)_3\cdot6H_2O$, 99.9%, ABCR), strontium nitrate ($Sr(NO_3)_2$, 99+%, Fisher), cobalt(II) nitrate hexahydrate ($Co(NO_3)_2\cdot6H_2O$, 98+%, ABCR), iron(III) nitrate nonahydrate ($Fe(NO_3)_3\cdot9H_2O$, 98%, ABCR), polyvinylpyrrolidone (M.W. = 10,000, ABCR), ethanol (99.8%, Fisher), polyvinyl butyral (Butvar B-98, Thermo Scientific), dibutyl phthalate (99%, Sigma), methyl ethyl ketone (99.5%, ROTH), NiO (Fiaxell SOFC Technologies), SDC ($Sm_{0.2}Ce_{0.8}O_{2-\delta}$, Sigma), ScCeSZ ($(Sc_2O_3)_{0.10}(CeO_2)_{0.01}(ZrO_2)_{0.89}$, Fiaxell SOFC Technologies), LSGM ($La_{0.8}Sr_{0.2}Ga_{0.2}Mg_{0.2}O_{3-\delta}$, 99%, Fiaxell SOFC Technologies), ScSZ ($(Sc_2O_3)_{0.06}(ZrO_2)_{0.94}$, Fiaxell SOFC Technologies), 8YSZ ($(Y_2O_3)_{0.08}(ZrO_2)_{0.92}$, Fiaxell SOFC Technologies), 3YSZ ($(Y_2O_3)_{0.03}(ZrO_2)_{0.97}$, Fiaxell SOFC Technologies), polymethyl methacrylate (PMMA, Fiaxell SOFC Technologies), gold paste (Lucerna), and carbon dioxide (99.9%, Carbagas) were all used as received without further purification.

Synthesis of Ni-SDC cathode

Ni-SDC was synthesized via a sol-gel process. Ethylenediaminetetraacetic acid (187.5 mmol) was first dissolved in 200 ml of 25wt% aqueous ammonia. To this solution, 100 mmol of $Ni(NO_3)_2\cdot6H_2O$, 20 mmol of $Ce(NO_3)_3\cdot6H_2O$, and 5 mmol of $Sm(NO_3)_3\cdot6H_2O$ were sequentially introduced. Citric acid (187.5 mmol) was then added as a chelating agent, and the pH was adjusted to 9 with additional aqueous ammonia. The mixture was maintained at 100°C under vigorous stirring overnight to evaporate water. Residual solvent was eliminated at 300°C for 20 h. The resulting precursor was grounded and calcined in air at 1,000°C for 6 h with a ramping rate of 5°C min^{-1}.

Synthesis of LSCF-SDC anode

LSCF-SDC was synthesized following a modified sol-gel process[1]. Ethylenediaminetetraacetic acid (65 mmol) was dissolved in 40 ml of 25wt% aqueous ammonia. Subsequently, 6 mmol of $La(NO_3)_3\cdot6H_2O$, 4 mmol of $Sr(NO_3)_2$, 2 mmol of $Co(NO_3)_2\cdot6H_2O$, 8 mmol of $Fe(NO_3)_3\cdot9H_2O$, 10 mmol of $Ce(NO_3)_3\cdot6H_2O$, and 2.5 mmol of $Sm(NO_3)_3\cdot6H_2O$ were added. Citric acid (65 mmol) was then introduced, and the solution pH

was adjusted to 8 by additional aqueous ammonia. The mixture was heated at 100°C under vigorous stirring for about 3 h to evaporate water, followed by drying at 300°C for 20 h. The dried precursor was grounded and calcined in air at 950°C for 5 h with a ramping rate of 10°C min^{-1}.

Preparation of slurry

The dispersant was prepared by dissolving polyvinylpyrrolidone (8.5 wt%) in ethanol. The binder solution contained polyvinyl butyral (20 wt%), dibutyl phthalate (10 wt%), methyl ethyl ketone (46 wt%), and ethanol (24 wt%). Each mixture was homogenized by roller milling at 70 rpm for 24 h. For slurry preparation, 28 g of powders were combined with 15 g of dispersant and 15 g of binder, followed by roller milling for at least 48 h.

Fabrication of HCSC

The HCSC (**Ni-ScSZ** | Ni-SDC | SDC | ScCeSZ | SDC | LSCF-SDC) was fabricated through sequential tape-casting, lamination and screen-printing steps (Figure S5). The support#1 slurry consisted of NiO (65 wt%), ScSZ (35 wt%), and PMMA (10 wt%). The support#2 slurry was prepared from a Ni-SDC cathode and polymethyl methacrylate (PMMA, 10 wt%) used as a pore former. Tapes for the support#2, support#1, cathode, buffer, and electrolyte were cast with raw thicknesses of 100, 1,200, 100, 30, and 30 μm, respectively, using a tape-casting machine (MTI Corporation), and dried at room temperature for 72 h. The dried tapes were laminated at 120°C using a hot laminator (Büro Tschudi), punched into a desired size, and sintered in air at 1,250°C for 6 h with a ramping rate of 2°C min^{-1}. The co-sintering temperature of 1,250°C was selected based on the sintering behavior of the constituent materials. Linear shrinkage measurements showed that the shrinkage of ScSZ and ScCeSZ approached a plateau at temperatures≥1,250°C (Figure S4), indicating that the sintering-induced shrinkage was essentially complete by this temperature. A second sintering, conducted under identical conditions with a 5 mm-thick alumina plate (AdValue Technology) placed on top of the cell, was performed to ensure cell flatness. The LSCF-SDC anode was subsequently screen-printed onto the buffer layer (Fiaxell SOFC technologies) and sintered in air at 1,050°C for 6 h at the same ramping rate. The support#1 layer was subsequently polished with sandpaper to obtain the final HCSC. The resulting cell had an active area of 1.0 cm^2. The thickness and mass of each layer were shown in Table S2.

Fabrication of MCSC

The MCSC (**Ni-ScSZ** || Ni-ScSZ || ScSZ || SDC || LSCF-SDC) was fabricated through sequential tape-casting, lamination and screen-printing steps (Figure S6). The support slurry was prepared from NiO (65 wt%), ScSZ (35 wt%), and PMMA (10 wt%) as a pore former. The cathode slurry consisted of NiO (65 wt%) and ScSZ (35 wt%). Tapes for the support, cathode, buffer, and electrolyte were cast with raw thicknesses of 1,200, 100, 30, and 30 μm, respectively, using a tape-casting machine, and dried at room temperature for 72 h. The dried tapes were laminated at 120°C using a hot laminator, punched into a desired size, and sintered in air at 1,250°C for 6 h with a ramping rate of 2°C min^{-1}. A second sintering, conducted under identical conditions with a 5 mm-thick alumina plate placed on top of the cell, was performed to ensure cell flatness. The LSCF-SDC anode was subsequently screen-printed onto the buffer layer and sintered in air at 1,050°C for 6 h at the same ramping rate. The final MCSC also had an active area of 1.0 cm^2. The thickness and mass of each layer were shown in Table S2.

Fabrication of ESC

The ESC (Ni-ScSZ || **ScSZ** || SDC || LSCF-SDC) was produced via a tape-casting process followed by multiple screen-printing processes (Figure S7). The ScSZ electrolyte slurry was tape-casted into sheets with a raw thickness of 1,000 μm, dried at room temperature for 72 h, then cut into shape and sintered in air at 1,400°C for 6 h with a ramping rate of 2°C min^{-1} to obtain a dense electrolyte support. A second sintering, conducted under identical conditions with a 5 mm-thick alumina plate placed on top of the electrolyte, was performed to ensure electrolyte flatness. The cathode slurry consisted of NiO (65 wt%) and ScSZ (35 wt%). The cathode and buffer layer were screen-printed onto both sides of the electrolyte support and sintered at 1,250°C for 6 h at 2°C min^{-1}. Finally, the LSCF-SDC anode was deposited by screen-printing, followed by sintering at 1,050°C for 6 h with the same ramping rate. The ESC also had an active area of 1.0 cm^2. The thickness and mass of each layer were shown in Table S2.

Mechanical strength measurements

Mechanical failure in practical devices most frequently occurs during cell fabrication and stack assembly, where components are subjected to handling, machining, and externally applied stresses at room temperature[2]. Under these conditions, resistance to crack initiation and propagation, effectively captured by room-temperature flexural strength, serves as a critical indicator of mechanical robustness. Moreover, although absolute strength values may decrease at elevated temperatures, it is widely recognized that the relative ranking of mechanical

strength among different materials and architectures is largely preserved[2,3]. Therefore, room-temperature flexural strength is adopted here as a practically relevant and comparative metric to evaluate intrinsic mechanical robustness.

Flexural strength was evaluated by a three-point bending test[4] at room temperature using a SHIMADZU EZ-test machine. For the ESC, MCSC, and HCSC, specimens were directly cut from the actual cells after reduction. For the different oxygen-ion conductors, species were separately prepared by tape casting (1 mm raw thickness), drying at room temperature for 72 h, and sintering in air at 1,400°C for 6 h with a ramping rate of 2°C min^{-1}. Rectangular bars (10 mm × 25 mm) were cut from the sintered pellets and tested on a fixture with a 20 mm support span at a crosshead speed of 2 mm min^{-1}. The flexural strength (σ_f) was calculated by equation (1):

$$\sigma_f = 3 \times F \times L / (2 \times W \times t^2) \quad (1)$$

where σ_f is flexural strength (MPa), F is fracture load (N), L is holder span (mm), W is sample width (mm), and t is thickness (mm).

Directly measured mechanical properties are generally unavailable for the literature-reported SOECs. We therefore estimated their flexural strength from the reported support thicknesses and flexural strength of the corresponding support materials using Weibull scaling relationship[3] by equation (2):

$$\sigma_{f2} = \sigma_{f1} \times (t_2 / t_1)^{-1/m} \quad (2)$$

where σ_{f2} is the strength of a sample with thickness of t_2, σ_{f1} is the reference strength at thickness t_1, and m is the Weibull modulus, assumed to be 12.[5] Using a cell size of 1 × 2 cm^2 and equation (1), we further calculated the corresponding fracture loads. Because cell fracture in practical devices depends on geometry, particularly thickness, we use fracture load rather than flexural strength as a more representative metric for comparing the mechanical robustness of different cell.

Conductivity measurements

Slurries were tape-casted into sheets with a raw thickness of 1 mm, dried at room temperature for 72 h, and sintered in air at 1,400°C for 6 h with a ramping rate of 2°C min^{-1}. The resulting pellets were cut into disk (Ø20 mm), coated on both sides with gold paste as current collectors, and fired at 850 C for 30 min to improve contact. The conductivity of oxides

was determined by EIS. EIS measurements were carried out under CO_2 atmosphere at open-circuit voltage with a perturbation amplitude of 10 mV over a frequency range of 100 kHz to 1 Hz. The ohmic resistance (R_o) was extracted from data fitting. For metallic samples, linear sweep voltammetry (LSV) was employed due to their low resistance. Samples were first reduced at 800°C for 30 min in H_2, then held at the target temperature under CO_2 during measurement. LSV were performed from 0 to 100 mA with a sweep rate of 1 mA s^{-1}, and R_o was determined from the slope of the LSV curve.

The conductivity (σ) was calculated by equation (3):

$$\sigma = t / (R_o \times A) \quad (3)$$

where σ is conductivity (S cm^{-1}), t is sample thickness (cm), R_o is ohmic resistance (Ω cm^2), and A is electrode area (cm^2).

Shrinkage measurements

Linear shrinkage was determined from dimensional changes during sintering. Tape-cast sheets (1 mm raw thickness) were dried at room temperature for 72 h and cut into 30 × 30 mm^2. The samples were sintered in air at the target temperature for 6 h with a ramping rate of 2°C min^{-1}, and the dimensions were measured with a vernier caliper. The shrinkage rate was calculated by equation (4):

$$\text{Shrinkage} = (l_i - l_0) / l_0 \quad (4)$$

where l_i and l_0 are the sample lengths after sintering and at room temperature, respectively (mm).

CO_2 electrolysis performance evaluation

Electrochemical performance was evaluated in a SOEC-V5 test station (Fiaxell SOFC Technologies) coupled to an Autolab potentiostat (PGSTAT302N) with a 20 A booster. The cell was heated to the desired temperature at a ramping rate of 5°C min^{-1} under air. Prior to testing, the cathode was activated by flowing 30 vol% H_2 in N_2 for 30 min. After reduction, pure CO_2 and air were supplied to the cathode and anode compartments, respectively, at 50 ml min^{-1}. The effluent from the cathode side was continuously analyzed by an on-line gas chromatograph (SRI instruments). Faradaic efficiency (FE) and energy efficiency (EE) were determined following previously reported procedures[11]. Electrochemical impedance

spectroscopy (EIS) was carried out at an applied voltage of 1.0 V using a 10 mV perturbation over a frequency range of 100 kHz to 0.01 Hz.

The scale-up measurements with an active cell area of 100 cm^2 were carried out in a SOEC-LCT test station (Fiaxell SOFC Technologies) coupled to a power supply PSI (0–60V/0–340A) using galvanostatic mode. The cell was heated to 700°C at a ramping rate of 5°C min^{-1} under air. Prior to testing, the cathode was activated by flowing 30 vol% H_2 in N_2 for 30 min. After reduction, pure CO_2 and air were supplied to the cathode and anode compartments, respectively, at 2,000 ml min^{-1}.

Characterizations

XRD patterns were recorded using a PANalytical Aeris Diffractometer with Cu *K*α radiation (40 kV, 15 mA). SEM images were acquired using a Zeiss GeminiSEM 300, with EDS mappings performed at an accelerating voltage of 16 kV. Raman spectra were collected using a Renishaw inVia confocal Raman microscope equipped with a 532 nm laser.

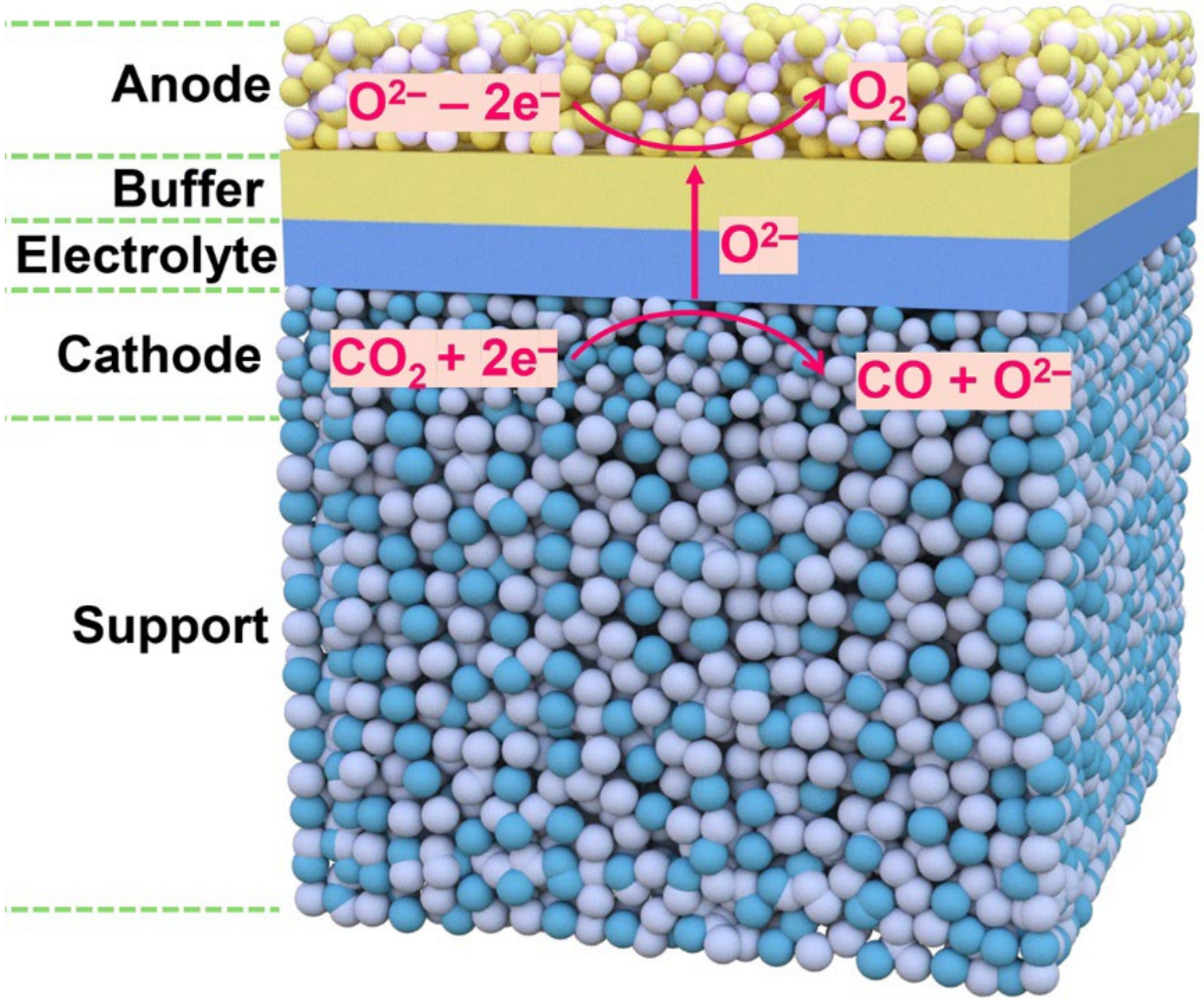

Figure S1. Schematic illustration of a typical cathode-supported cell for high-temperature CO_2-to-CO conversion. Gray balls represent Ni. All other colored balls or plates represent oxide phases, with each color corresponding to a distinct oxide.

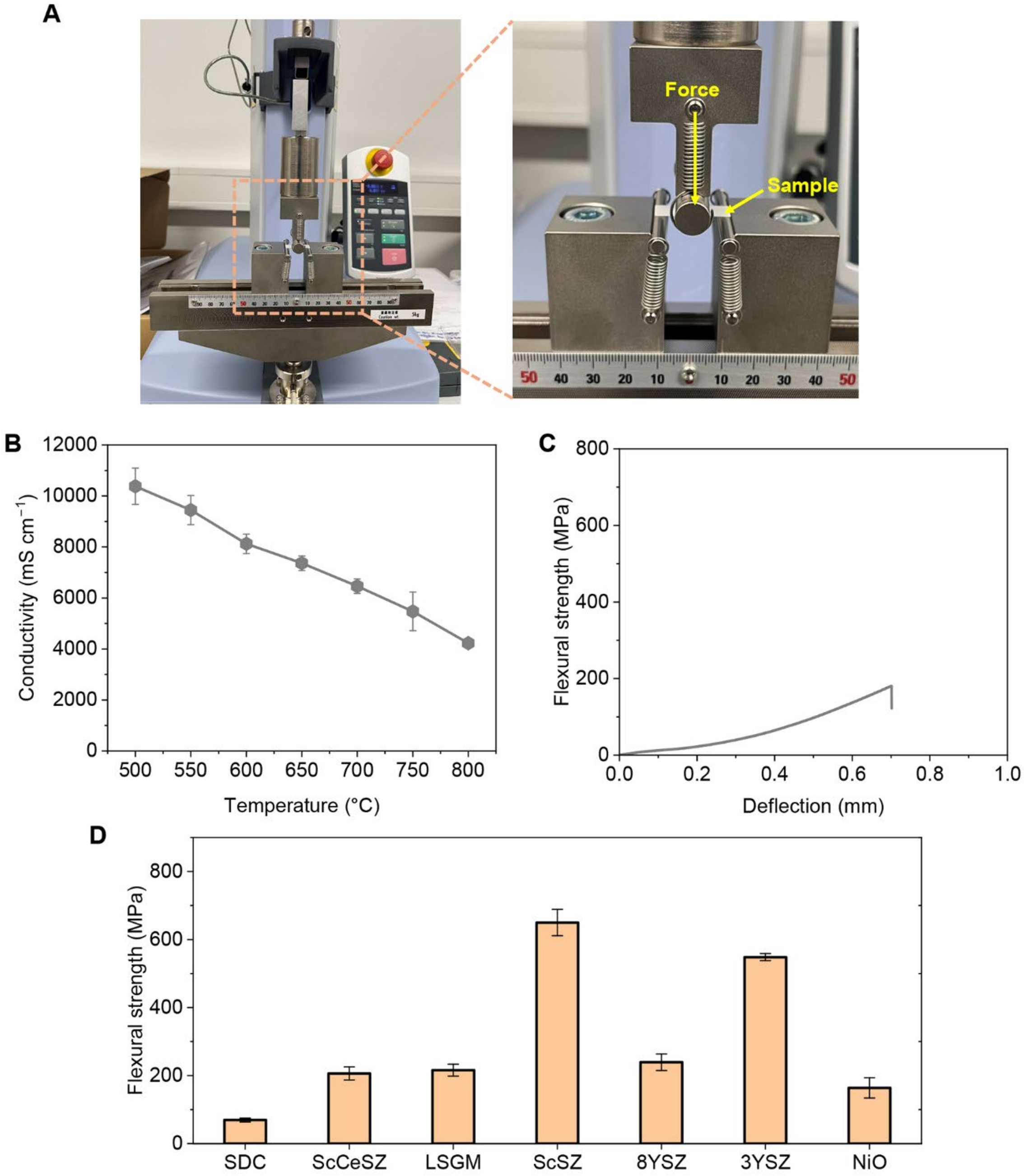

Figure S2. Flexural strength and conductivity for different materials. (A) Photographs of the three-point bending test station. (B) Conductivity of NiO after reduction to its metallic form. (C) Flexural strength-deflection curves of NiO in its oxide form. (D) Comparison of flexural strengths among different materials. The results are shown as the mean ± s.d. from three individual experiments.

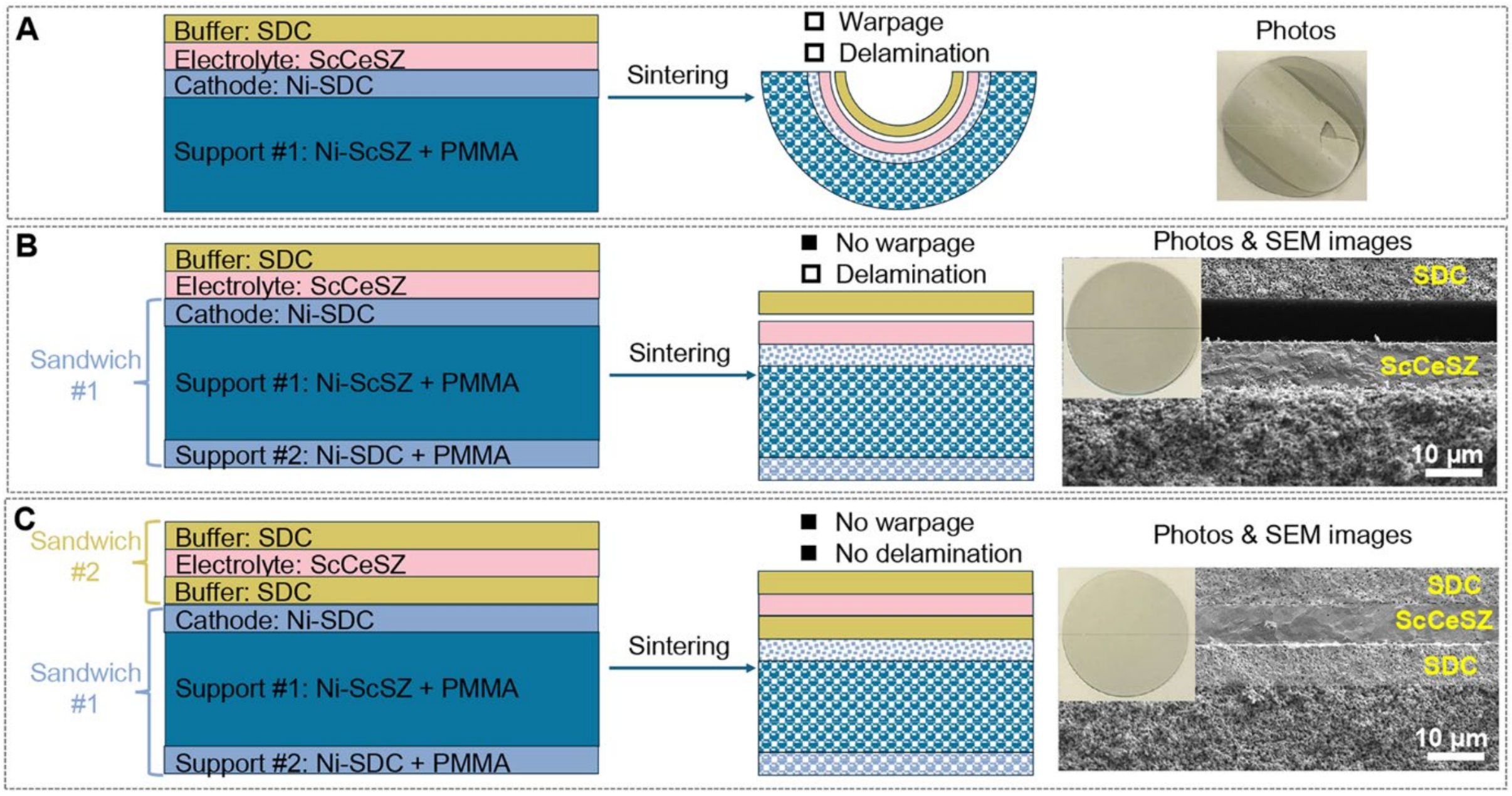

Figure S3. Sandwich-structured half-cell designs addressing warpage and delamination issues. (A) A Ni-ScSZ | Ni-SDC | ScCeSZ | SDC structure exhibits significant warpage and delamination. (B) A Ni-SDC | Ni-ScSZ | Ni-SDC | ScCeSZ | SDC structure mitigates warpage but still shows delamination. (C) A Ni-SDC | Ni-ScSZ | Ni-SDC | SDC | ScCeSZ | SDC structure effectively eliminates both warpage and delamination.

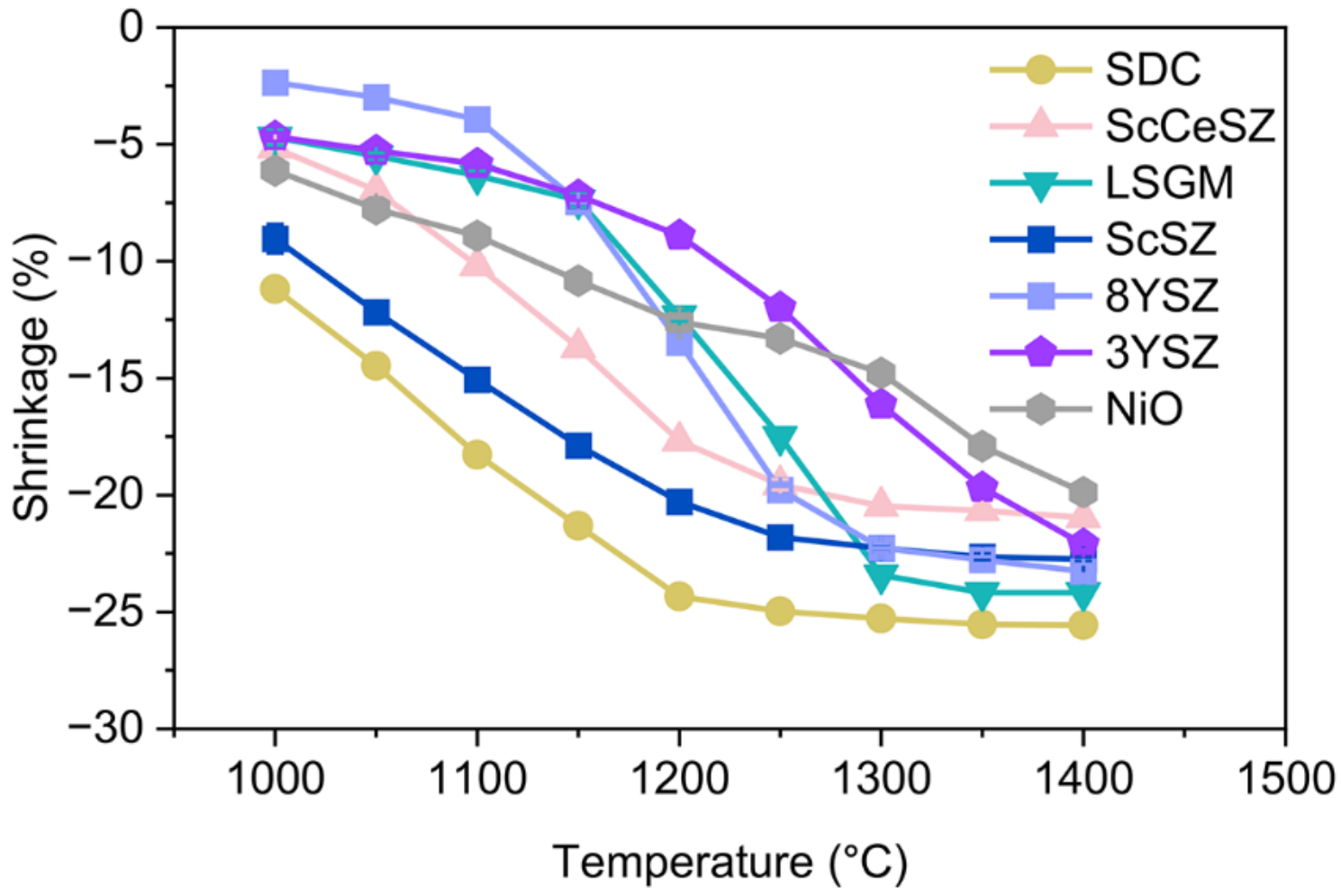

Figure S4. Linear shrinkage as a function of temperature for different materials. The results are shown as the mean ± s.d. from three individual experiments.

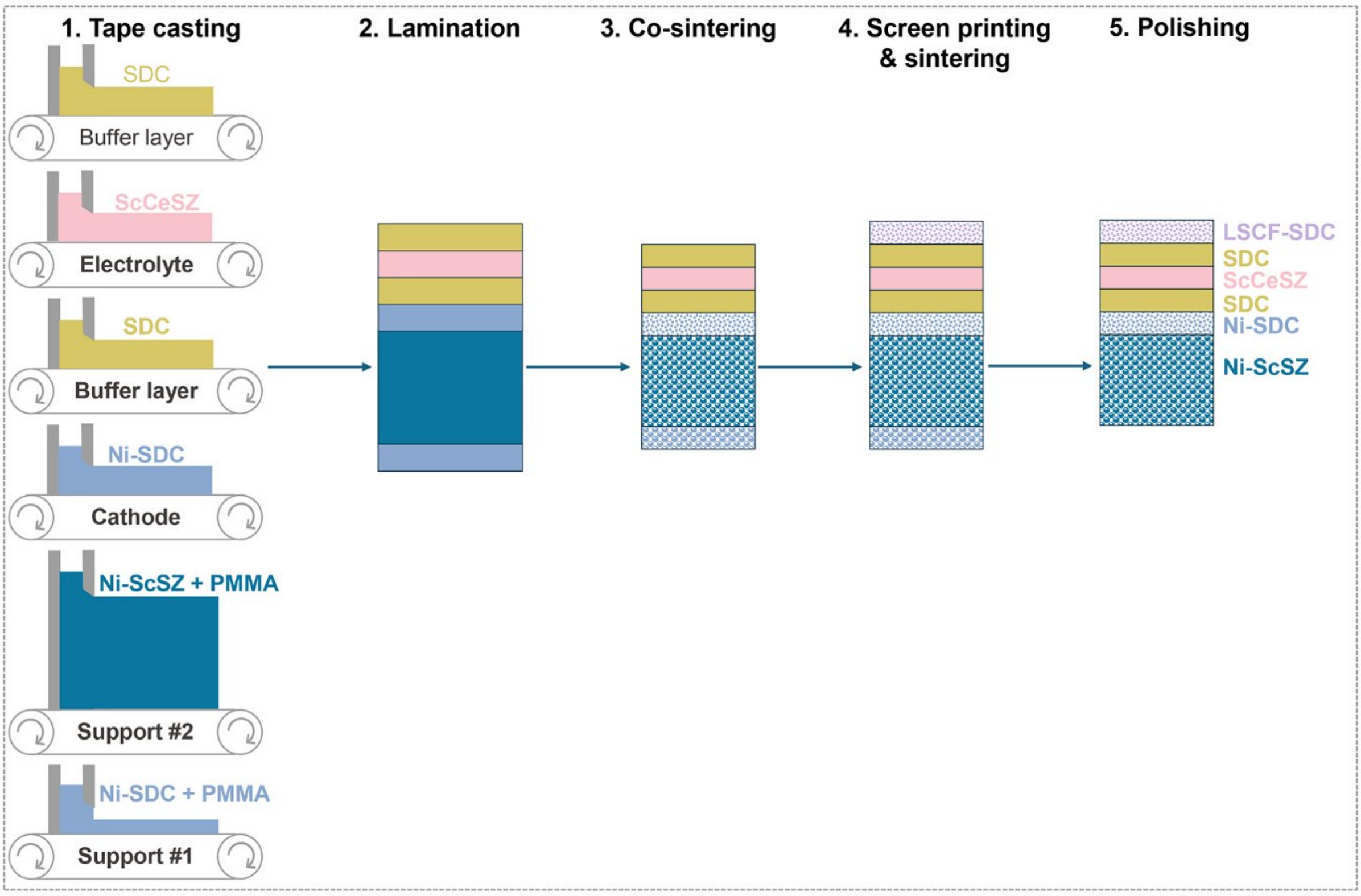

Figure S5. Schematic illustration of the fabrication process of the HCSC.

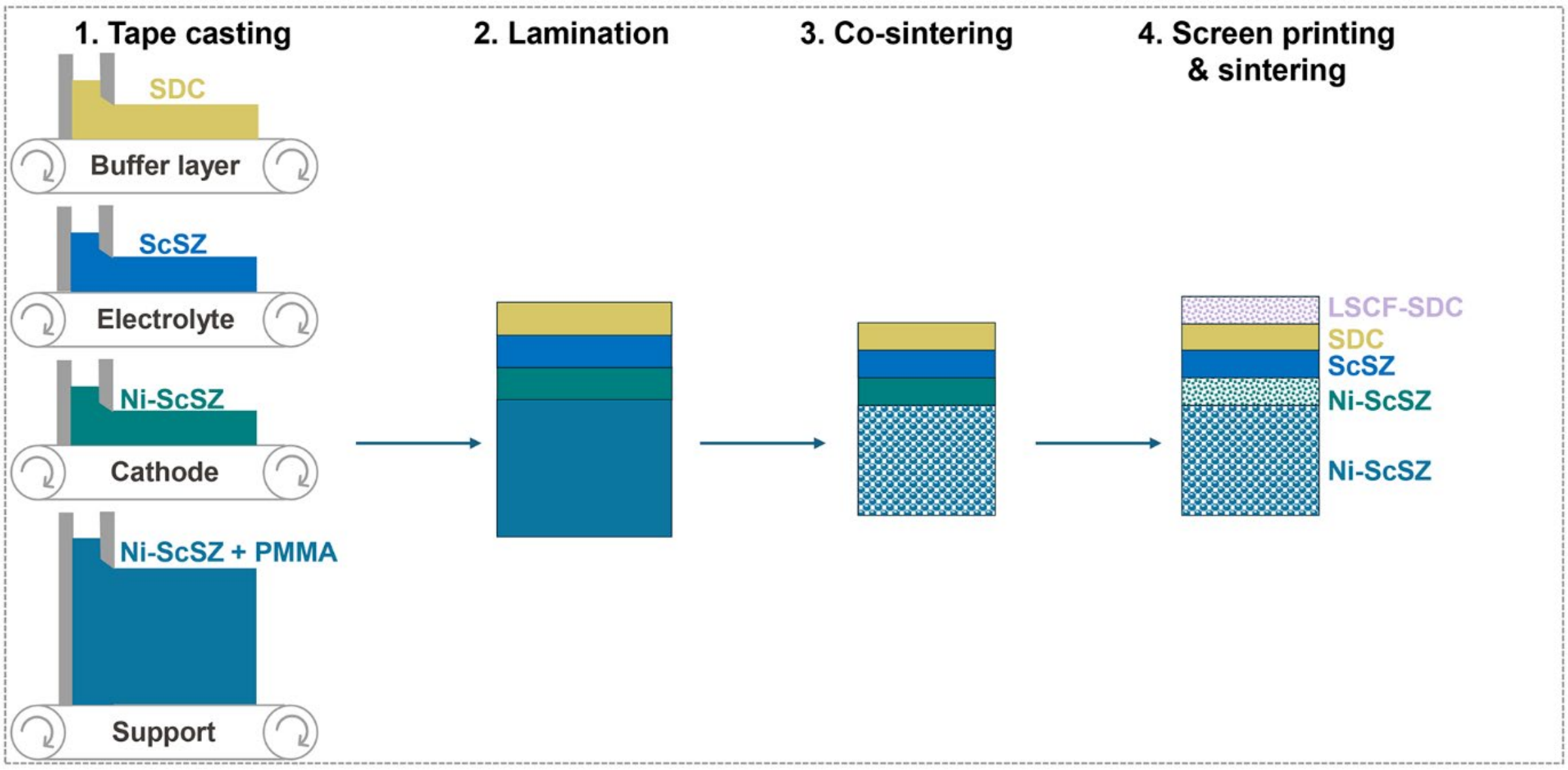

Figure S6. Schematic illustration of the fabrication process of the MCSC.

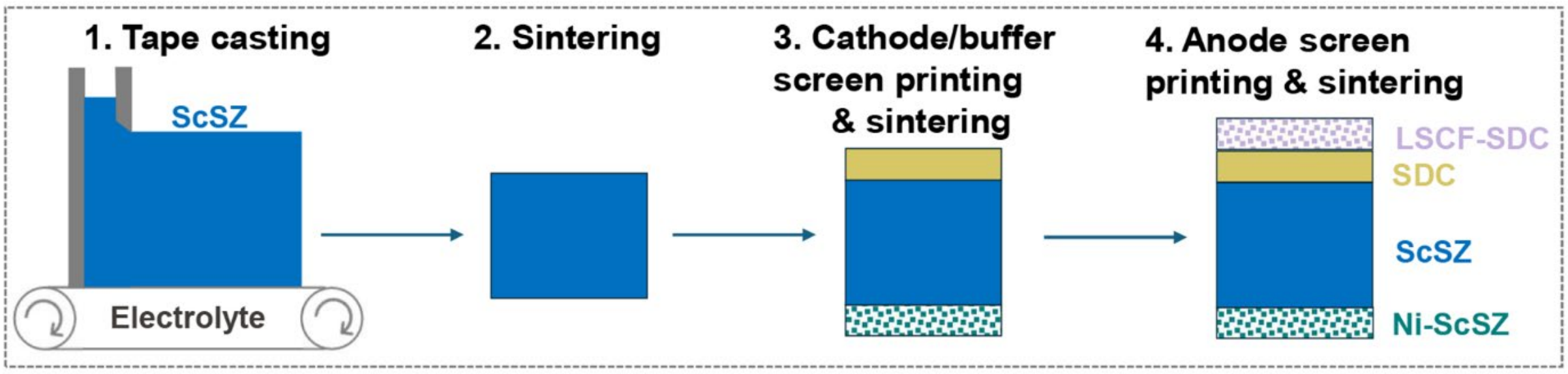

Figure S7. Schematic illustration of the fabrication process of the ESC.

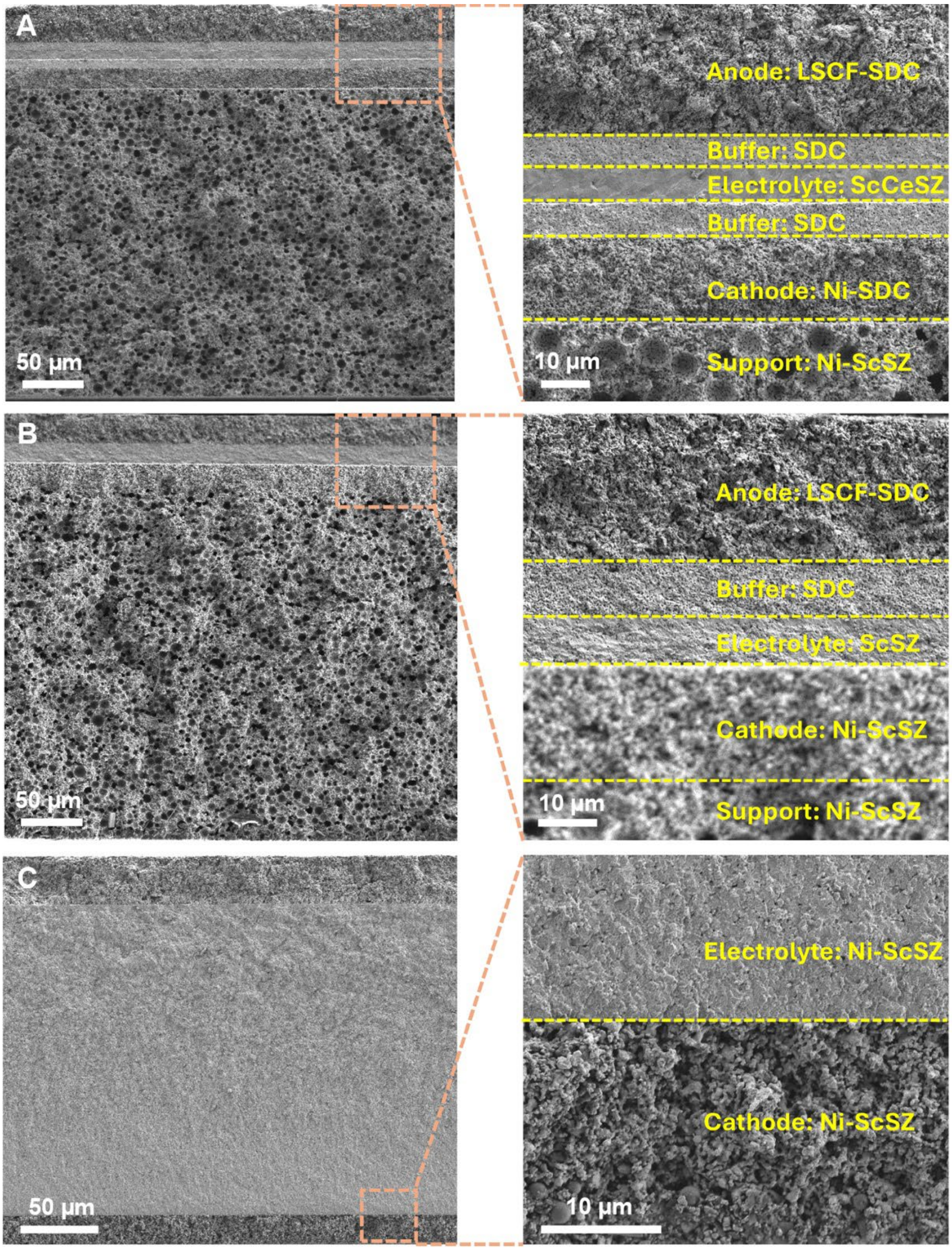

Figure S8. Cross-sectional SEM images of fresh cells. (A) HCSC. (B) MCSC. (C) ESC.

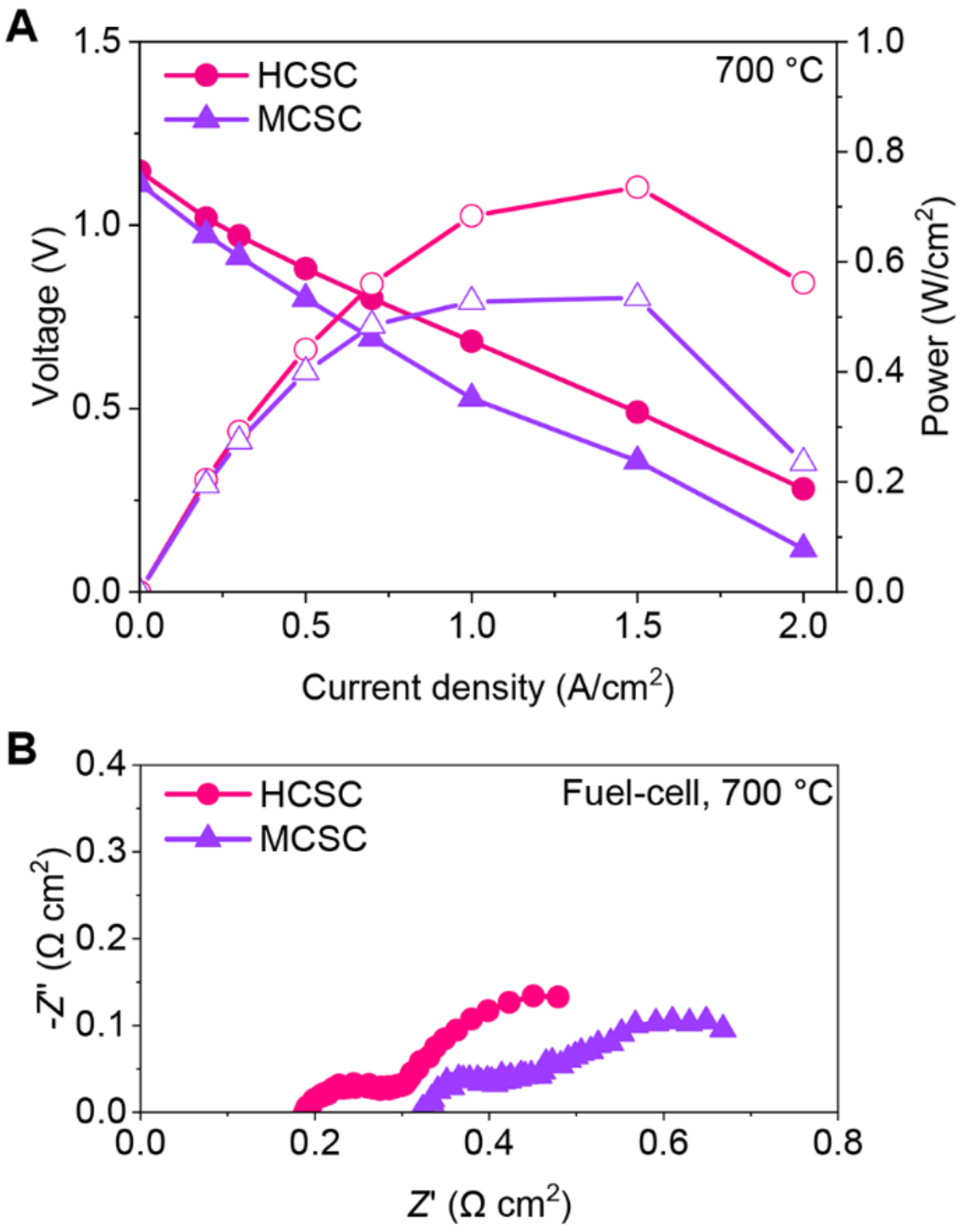

Figure S9. H_2–O_2 fuel cell performance of the HCSC and MCSC at 700℃. (A) Polarization and power density curves. (B) EIS spectra at 0.1 A. The anode and cathode were supplied with 50%H_2/N_2 and air, respectively, each at 50 ml/min.

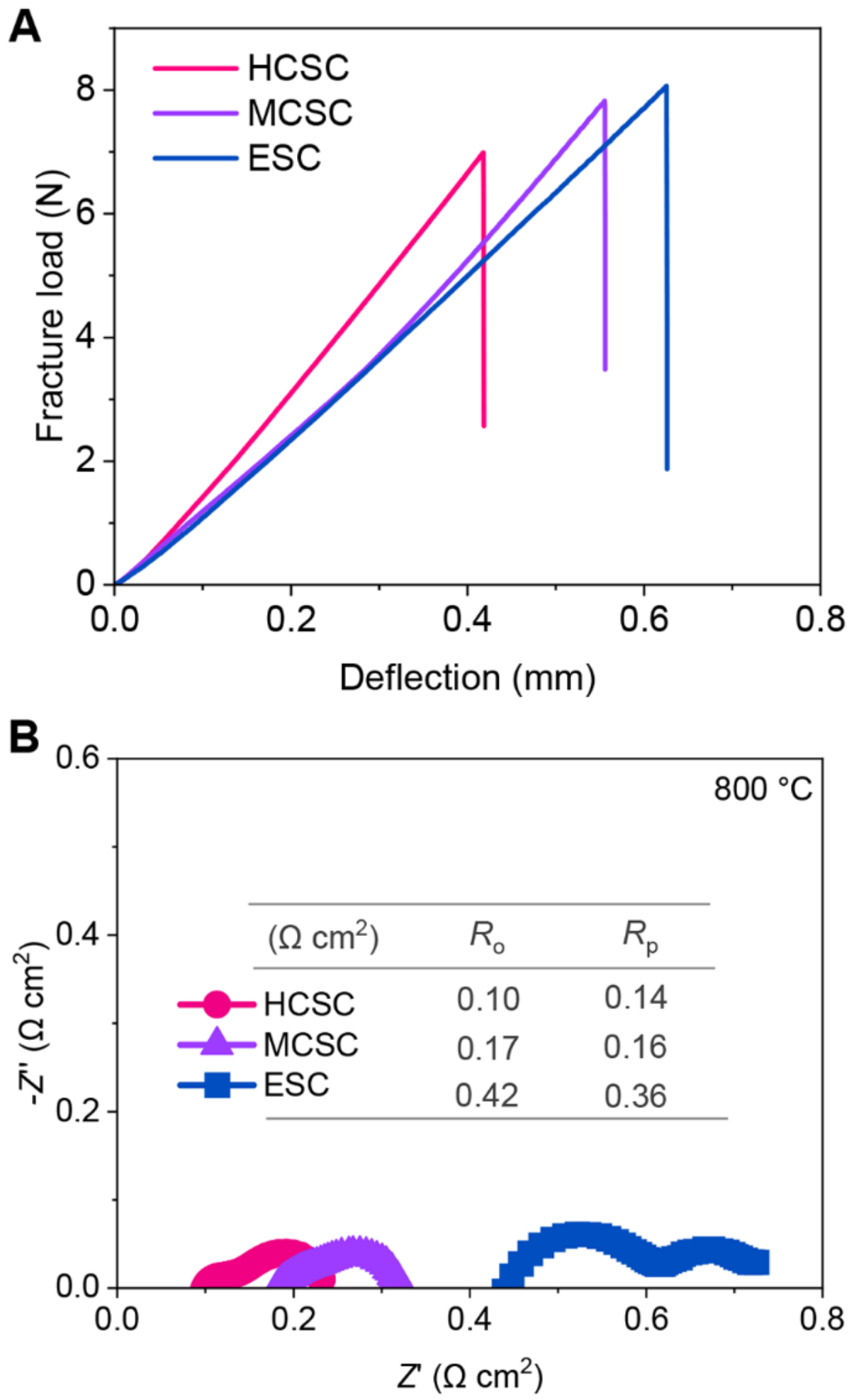

Figure S10. Fracture-deflection curves and EIS spectra of different cells. (A) Fracture load-deflection curves. (B) EIS spectra at 800°C.

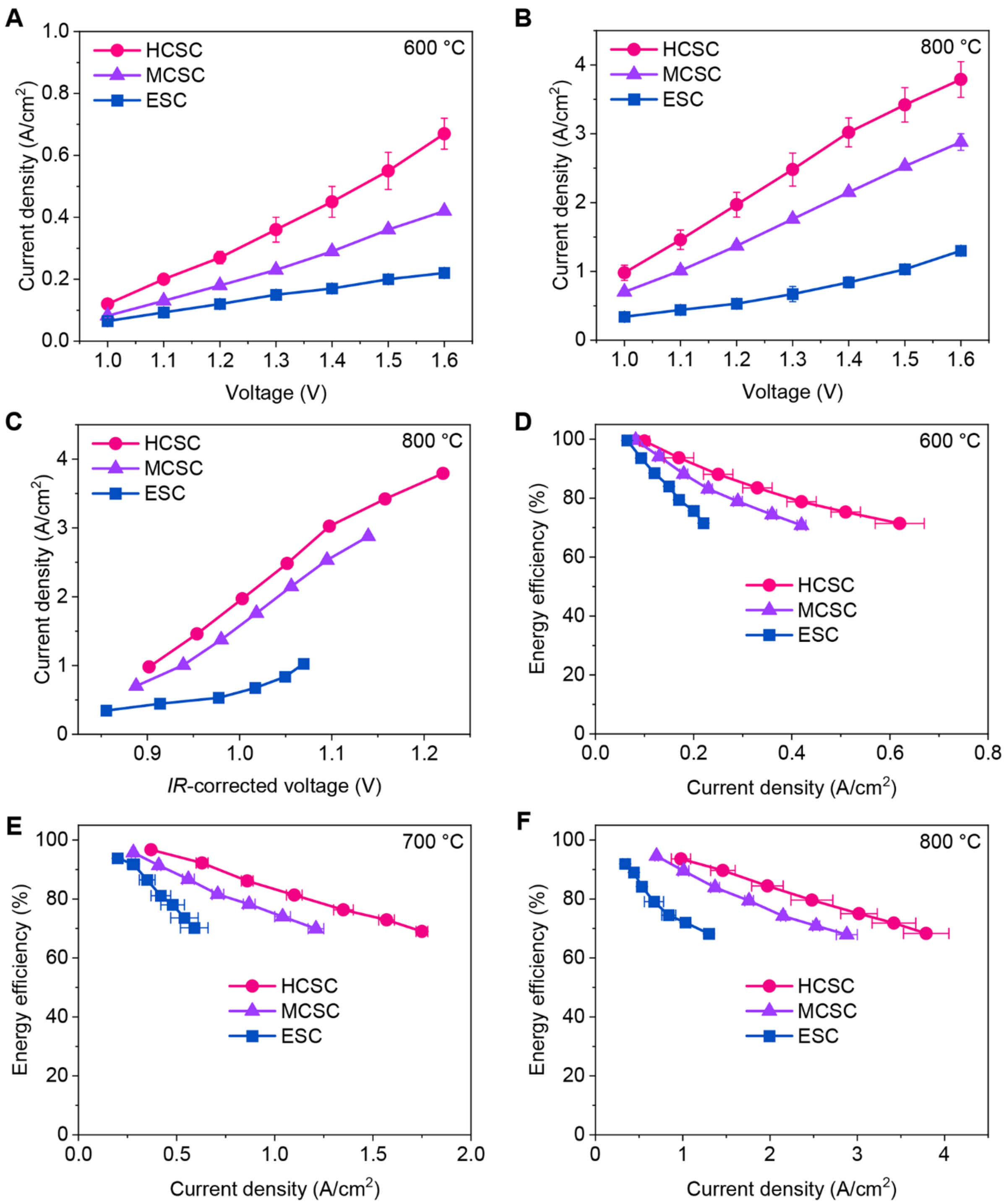

Figure S11. Activity and energy efficiency of CO_2 electrolysis at different temperatures. (A) Current density-voltage curves of different cells at 600°C. (B) Current density-voltage curves of different cells at 800°C. (C) *IR*-corrected performance of different cells at 800°C. (D) Energy efficiency as a function of current density for different cells at 600°C. (E) Energy efficiency as a function of current density for different cells at 700°C. (F) Energy efficiency as a function of current density for different cells at 800°C. The results are shown as the mean ± s.d. from three individual experiments.

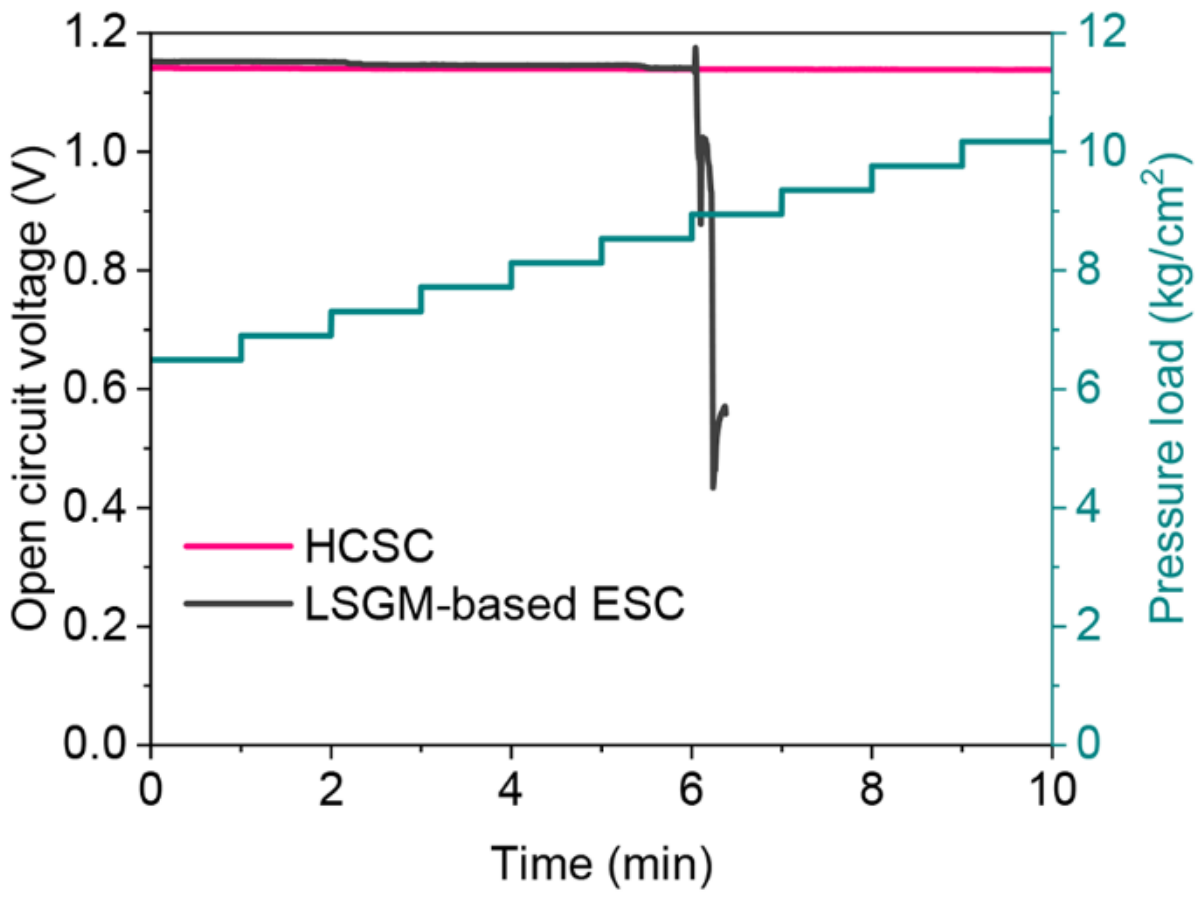

Figure S12. Pressure-loading stability of the HCSC and LSGM-based ESC under operating conditions. The LSGM-based ESC was fabricated following our previously reported procedure[1]. The anode and cathode were supplied with 50%H_2/N_2 and air, respectively, each at 50 ml/min. The measurements were performed at 700°C with progressively increasing pressure loads.

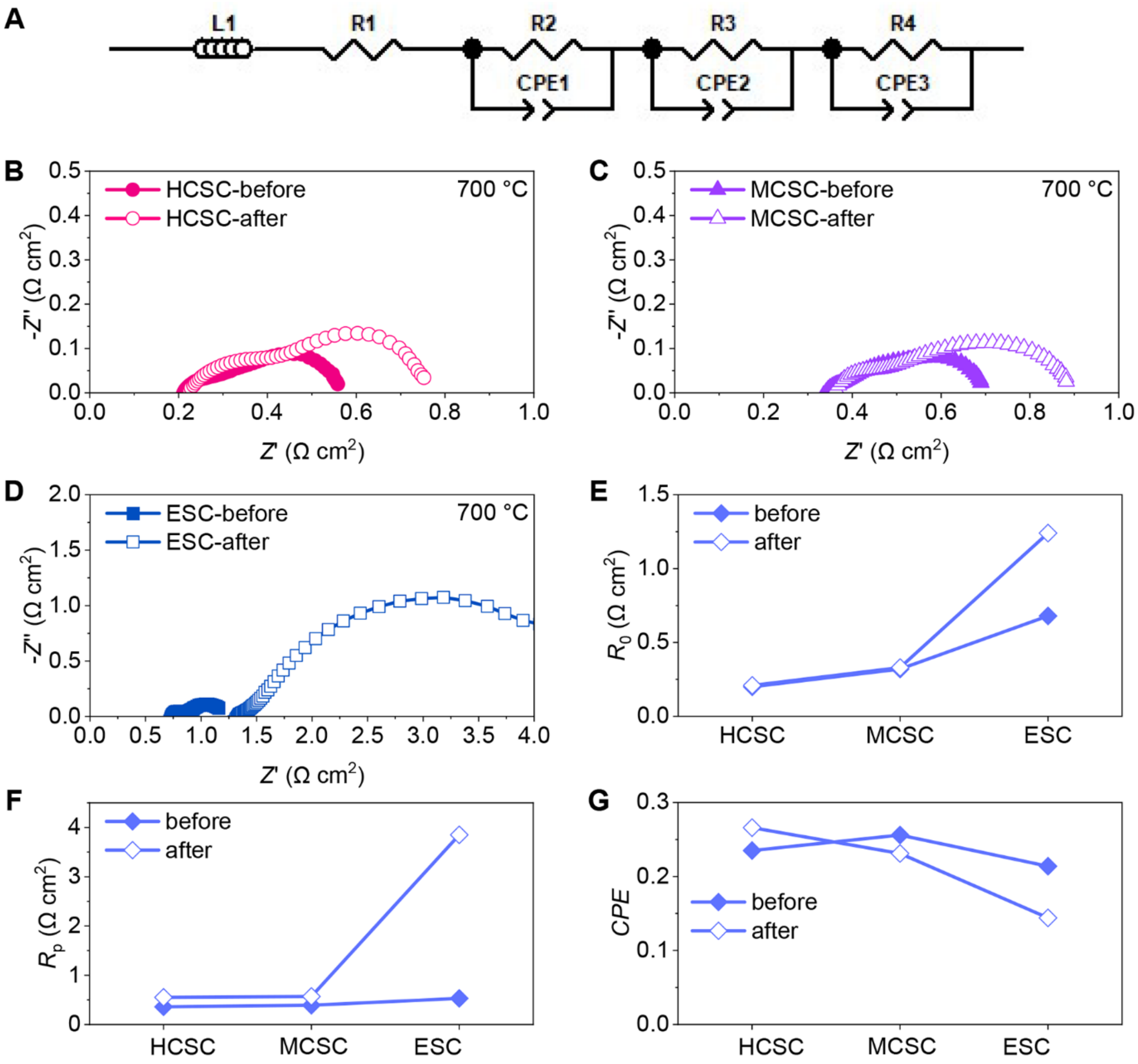

Figure S13. EIS spectra and fitted results of different cells before and after stability testing. (A) Equivalent circuit model used for EIS fitting. (B) EIS spectra of the HCSC at 1.0 V and 700°C. (C) EIS spectra of the MCSC at 1.0 V and 700°C. (D) EIS spectra of the ESC at 1.0 V and 700°C. (E) Fitted ohmic resistances. (F) Fitted polarization resistances (R_p). (G) Fitted constant phase element (*CPE*). R_o corresponds to R_1, and R_p is calculated as $R_p = R_2 + R_3 + R_4$. The overall *CPE* value is determined by $1/CPE = 1/CPE_1 + 1/CPE_2 + 1/CPE_3$.

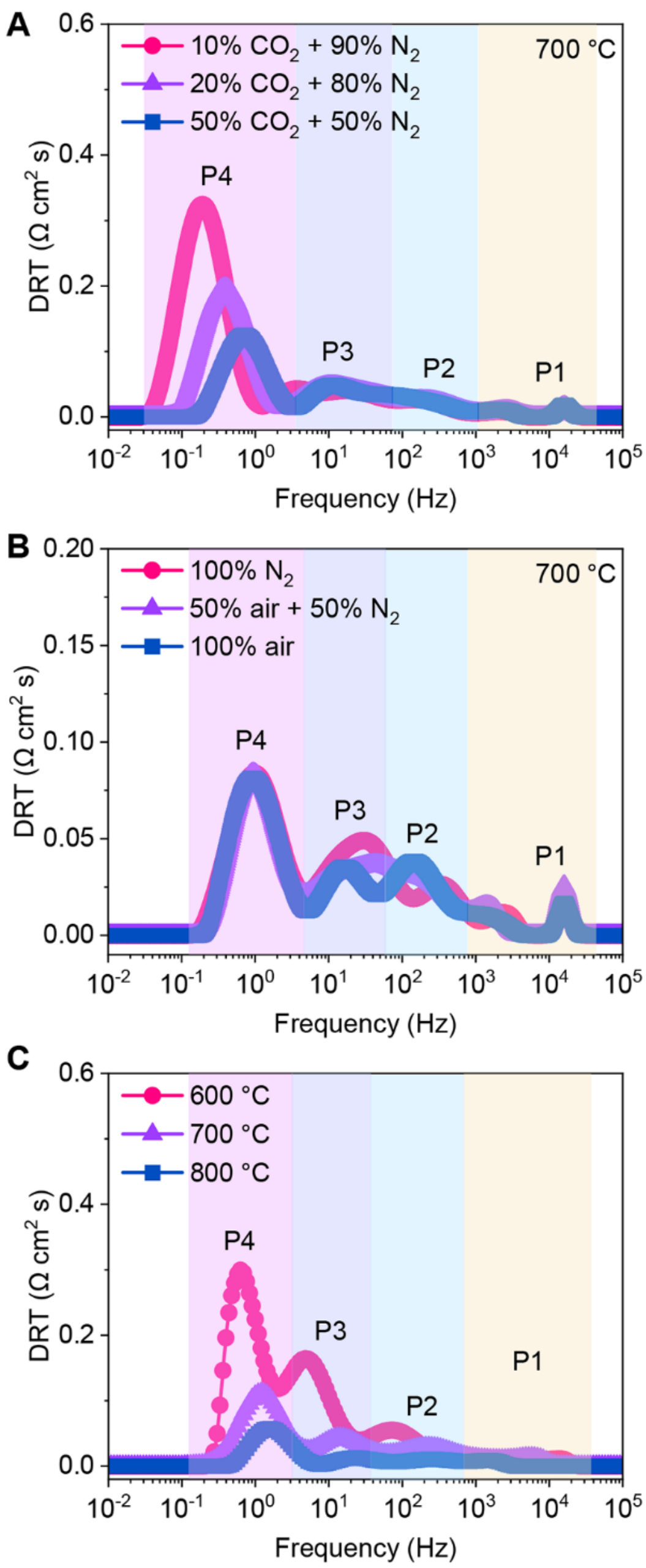

Figure S14. DRT analyses of the HCSC under varying operating conditions. (A) Effect of cathode CO_2 concentration at 1.0 V and 700°C. The total cathode gas flow rate was maintained at 50 ml/min, with N_2 used to dilute CO_2, while the anode was supplied with pure N_2 at 50 ml/min. (B) Effect of anode O_2 concentration at 1.0 V and 700°C. The total anode gas flow rate was maintained at 50 ml/min, with N_2 used to dilute air, while the cathode was supplied with pure CO_2 at 50 ml/min. (C) Effect of operating temperature at 1.0 V. The cathode and anode were supplied with CO_2 and air, respectively, each at 50 ml/min.

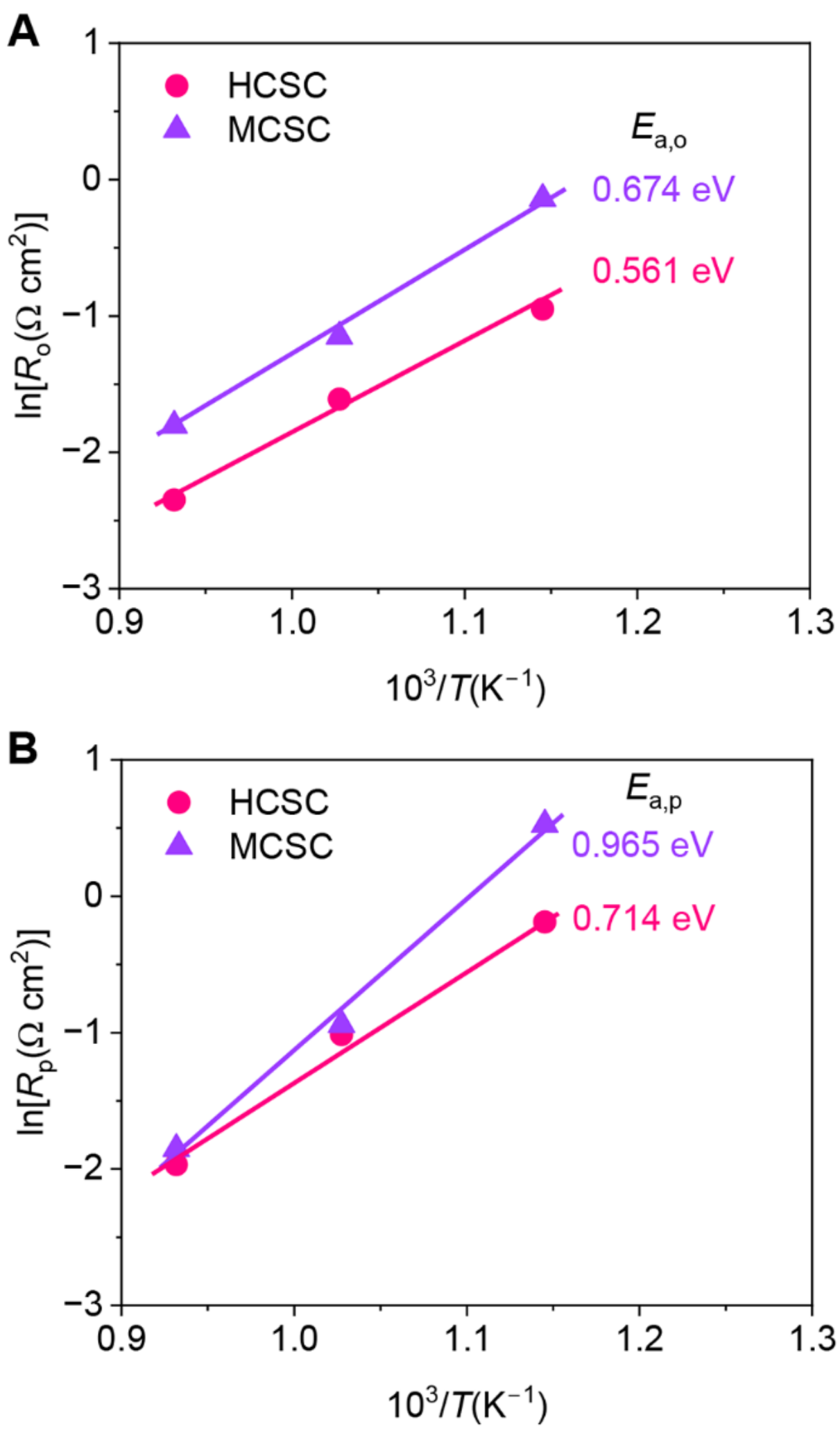

Figure S15. Arrhenius plot of ohmic and polarization resistance for different cells. (A) Arrhenius plot of ohmic resistance (R_o) and the calculated ohmic activation energy ($E_{a,o}$). (B) Arrhenius plot of polarization resistance (R_p) and the calculated polarization activation energy ($E_{a,p}$). R_o and R_p follow an Arrhenius-type temperature dependence, expressed as $\ln R = E_a/(k_B T) + C$, where k_B is the Boltzmann constant (8.617×10^{-5} eV/K), T is the absolute temperature (K), and C is a constant. Accordingly, a plot of ln$R$ versus $1/T$ yields a linear relationship, with the slope corresponding to E_a/k_B.

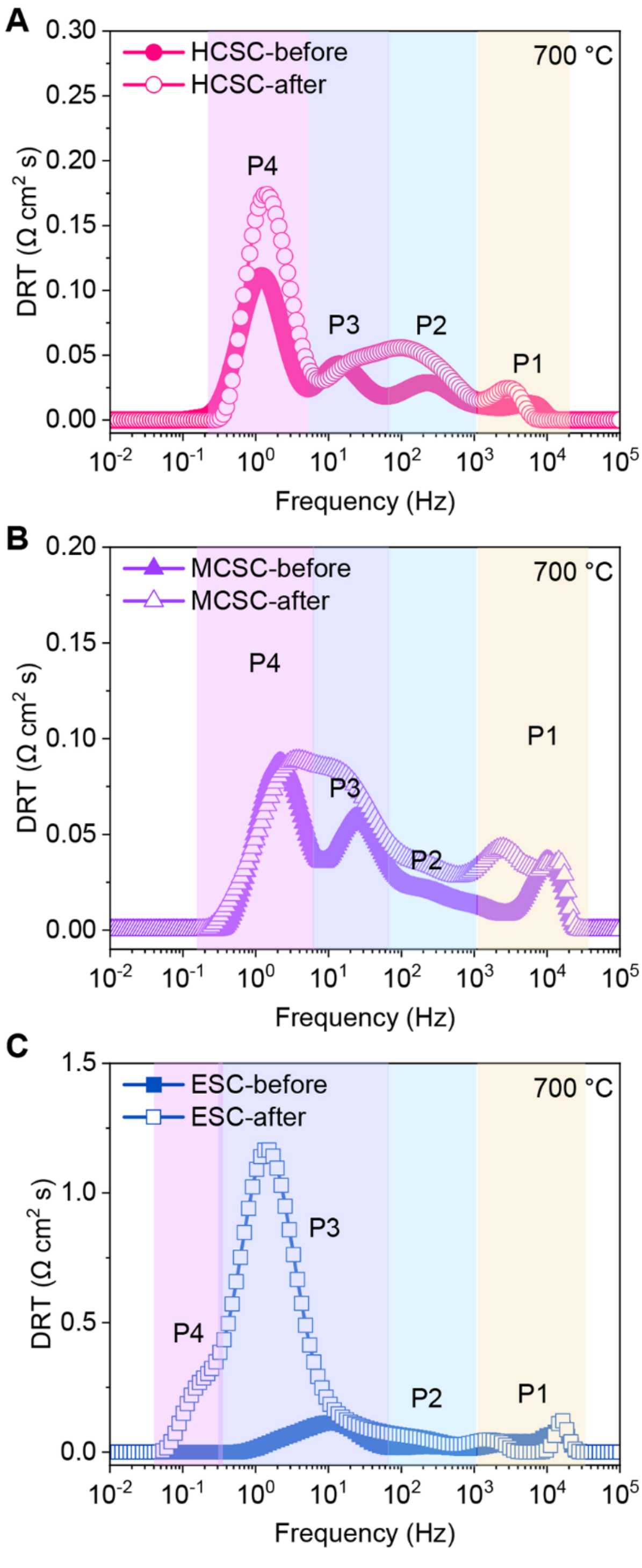

Figure S16. DRT analyses of different cells before and after stability testing. (A) The HCSC at 1.0 V and 700°C. (B) The MCSC at 1.0 V and 700°C. (C) The ESC at 1.0 V and 700°C.

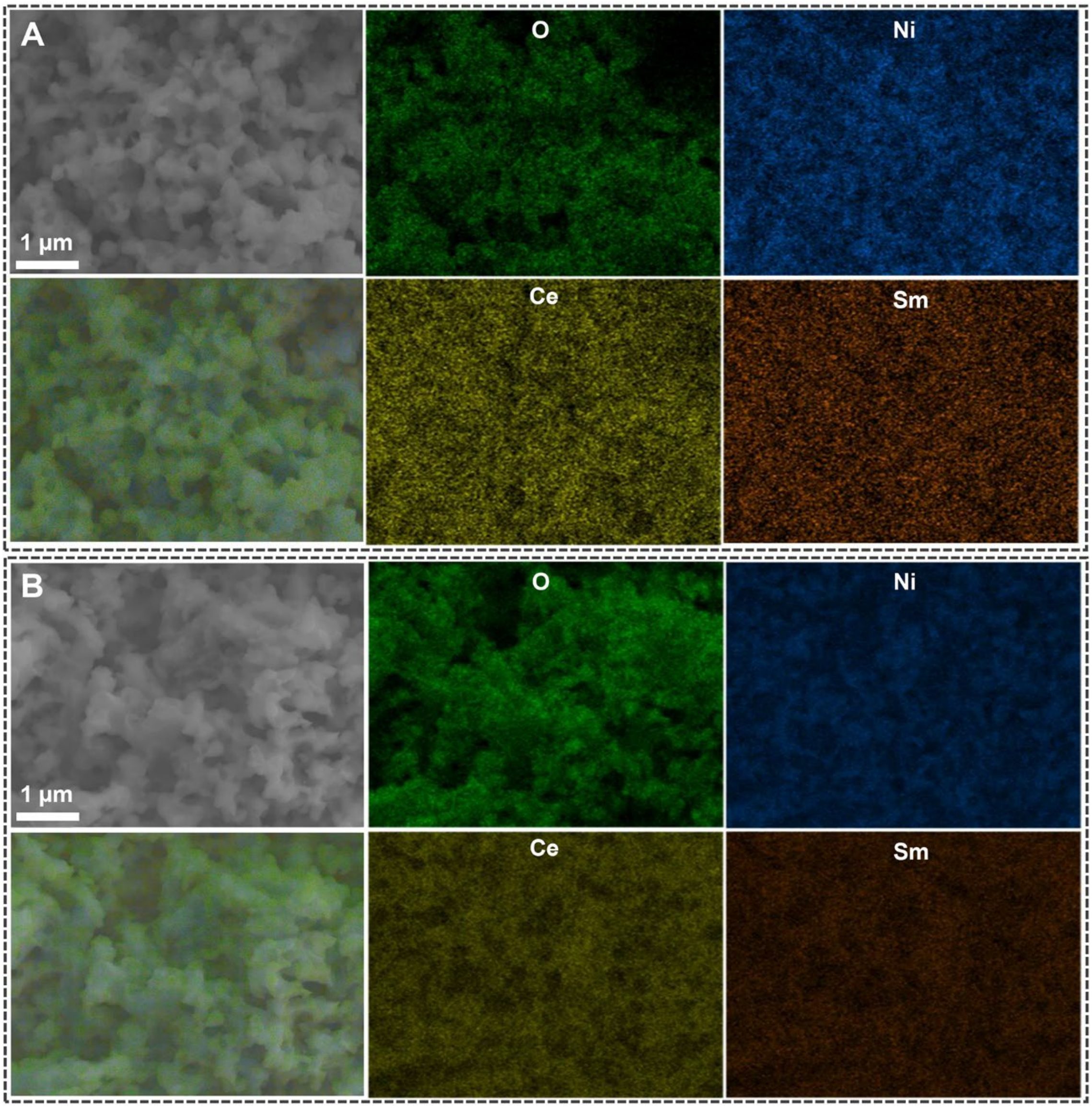

Figure S17. SEM images and the corresponding EDX mappings of the Ni-SDC cathode in HCSC. (A) Before stability. (B) After stability.

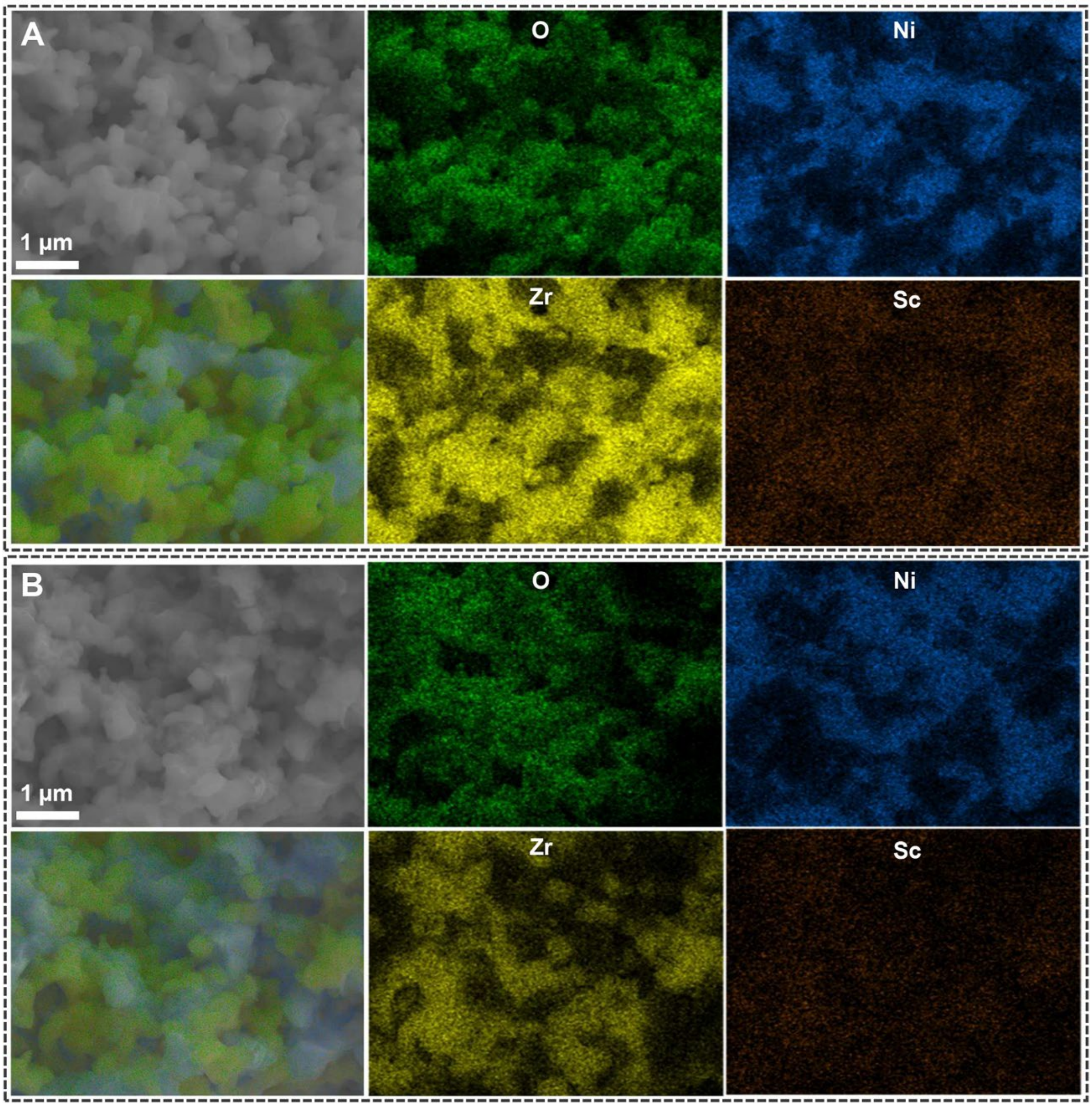

Figure S18. SEM images and the corresponding EDX mappings of the Ni-ScSZ cathode in MCSC. (A) Before stability. (B) After stability.

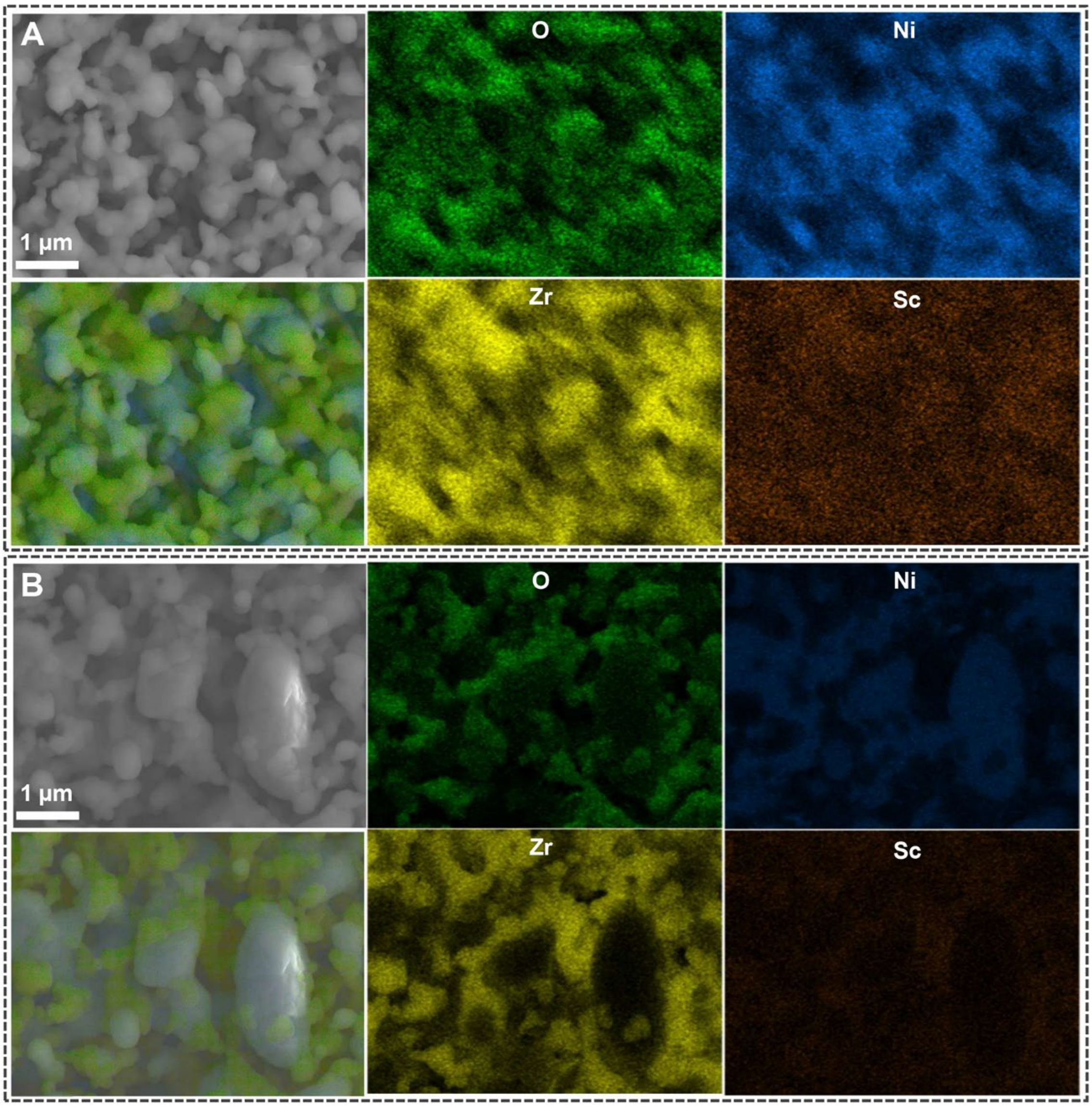

Figure S19. SEM images and the corresponding EDX mappings of the Ni-ScSZ cathode in ESC. (A) Before stability. (B) After stability.

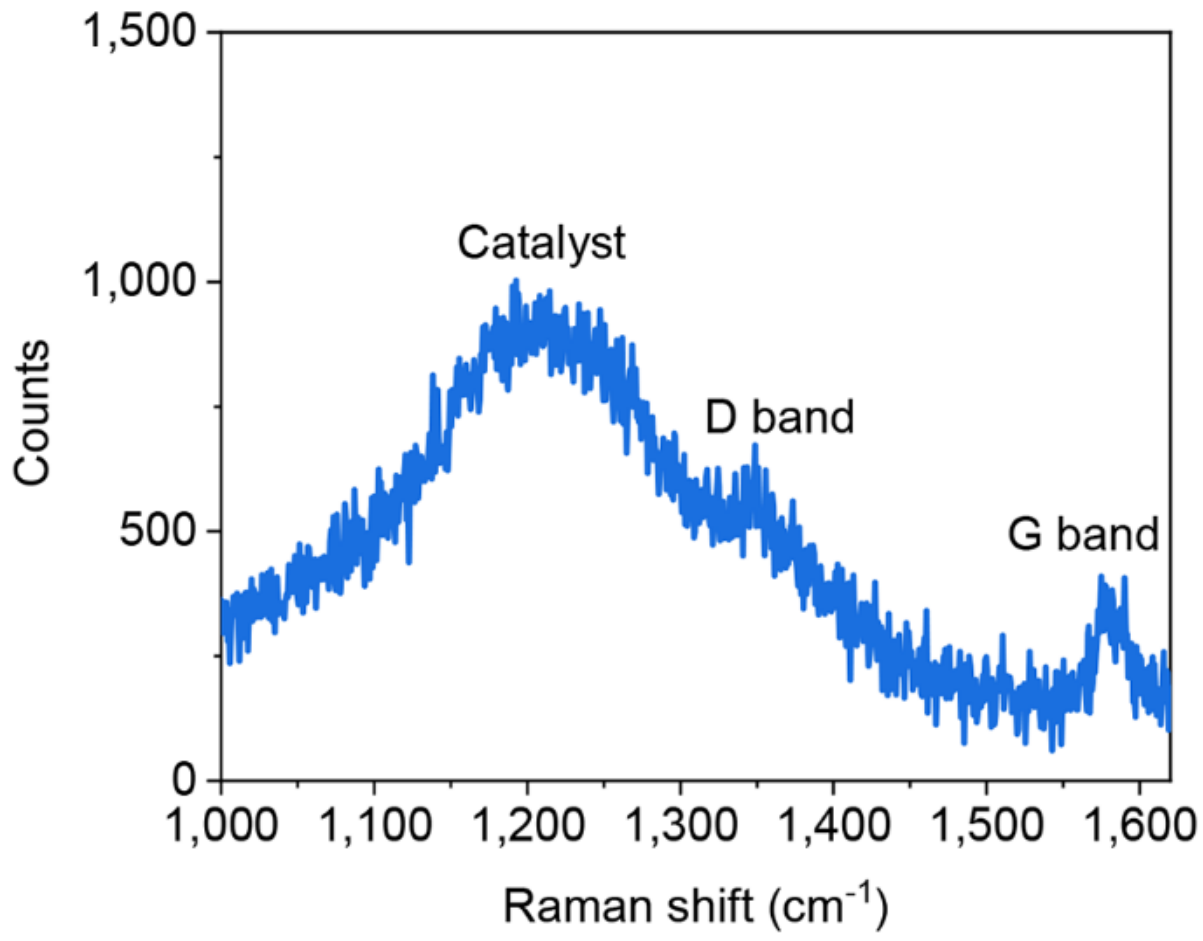

Figure S20. Raman spectrum of the Ni-SDC cathode in the HCSC after stability testing.

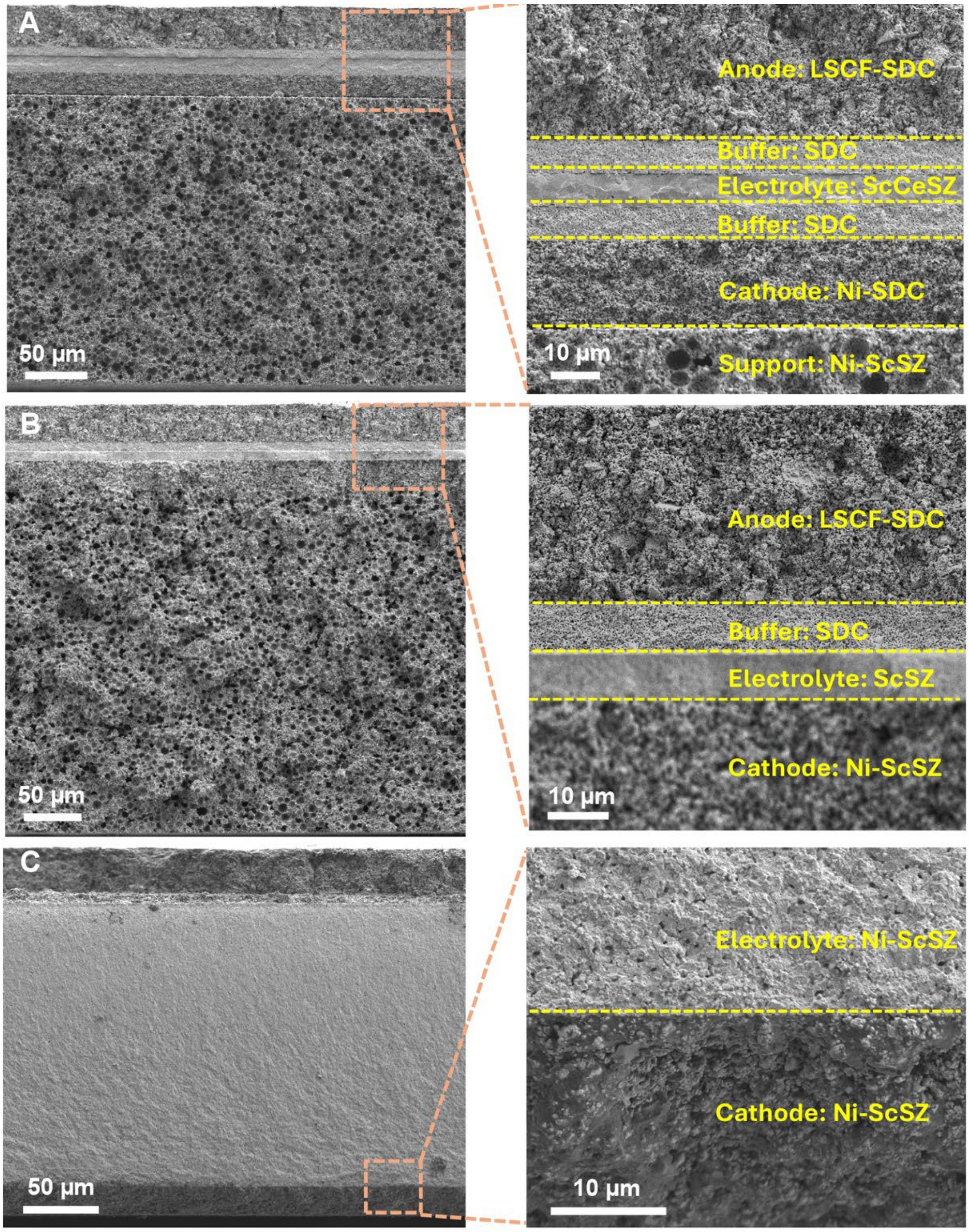

Figure S21. Cross-sectional SEM images of different cells after stability testing. (A) HCSC. (B) MCSC. (C) ESC.

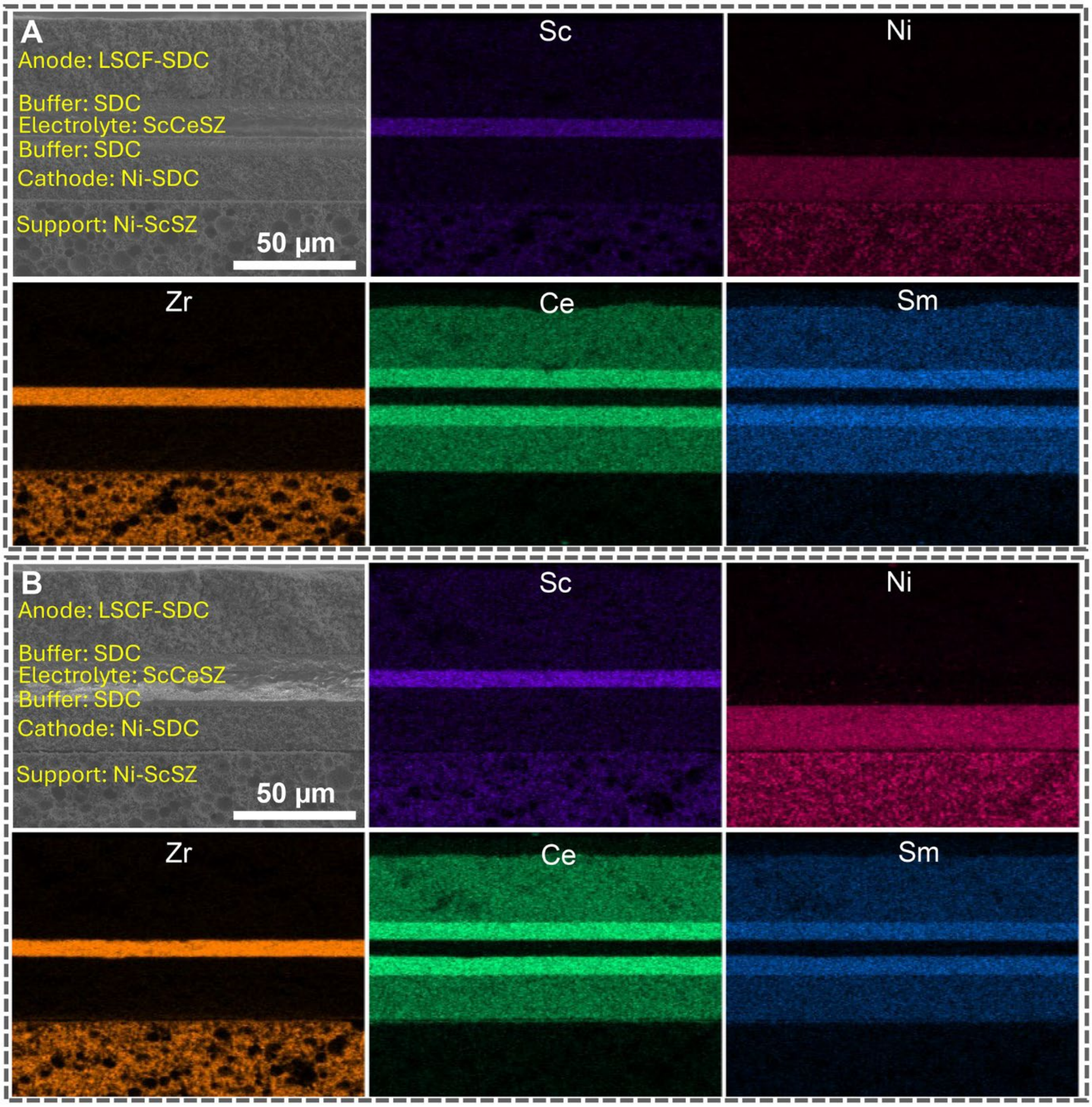

Figure S22. Cross-sectional SEM-EDS mappings of the HCSC before and after stability testing. (A) Before stability testing. (B) After stability testing.

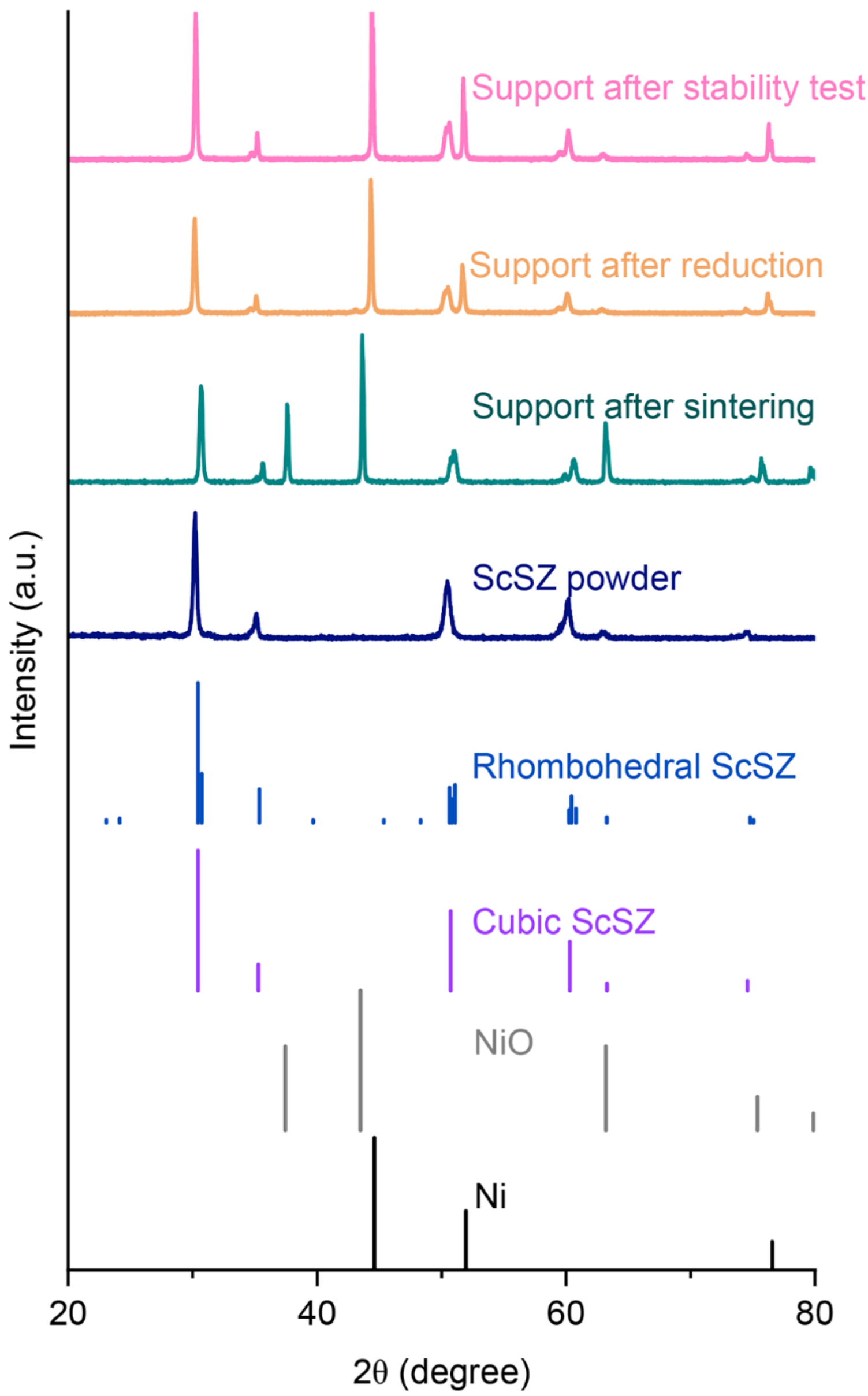

Figure S23. XRD patterns of support layers in different states. Ni: JCPDS, 96-901-2968; NiO: JCPDS, 00-001-1239; Cubic ScSZ: JCPDS, 98-008-6709; Rhombohedral ScSZ: JCPDS, 00-035-1492; ScSZ powder: as-received ScSZ powder without any treatment; Support after sintering: NiO-ScSZ support layer after sintering at 1,250°C; Support after reduction: Ni-ScSZ support layer after reduction; Support after stability: Ni-ScSZ support after stability test.

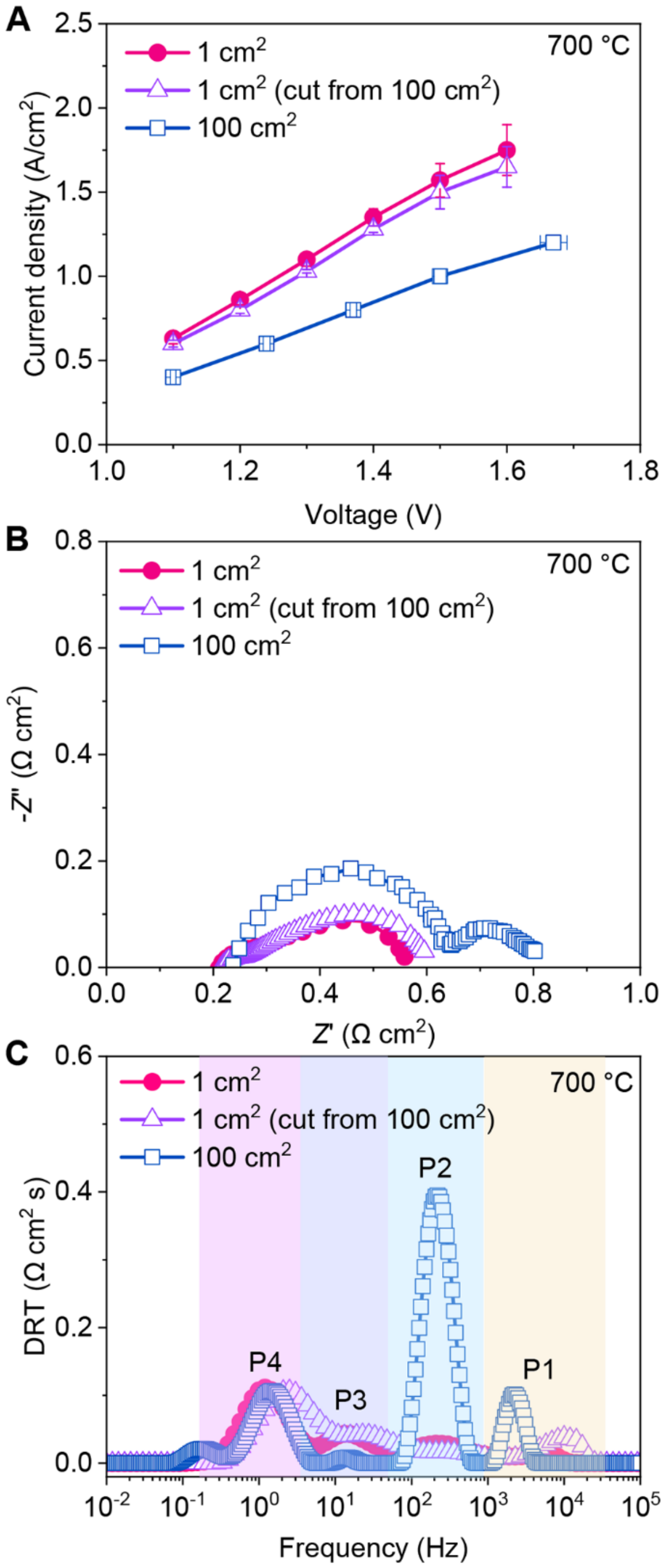

Figure S24. Electrochemical measurements of the 100 cm^2 HCSC at 700°C. (A) Current density-voltage curves. (B) EIS spectra. (C) DRT analyses.

Table S1. Comparison of our cell with representative SOECs.

Cell	Support thickness (μm)	Fracture (N)	Current density at 1.3 V (A/cm^2)	Stability (h)	Ref.
LCN-GDC | **8YSZ** | LCN-GDC (ESC)	200	3.2	0.25 at 700°C<br>0.7 at 800°C	120<br>(0.7 A/cm^2)	6
NMC-GDC | **8YSZ** | NMC-GDC (ESC)	200	3.2	0.25 at 700°C<br>0.5 at 800°C	70<br>(0.8 A/cm^2)	7
LCFNb-ScSZ | **ScSZ** | LSM-ScSZ (ESC)	125	4.1	0.2 at 700°C<br>0.4 at 800°C	10<br>(0.9 A/cm^2)	8
Ca-LSCM-GDC | **ScSZ** | LSM-GDC (ESC)	200	10.1	0.14 at 700°C<br>0.3 at 800°C	100<br>(0.23 A/cm^2)	9
Co-GDC | **ScSZ** | GDC | LSM (ESC)	116	3.6	0.24 at 800°C	48<br>(0.2 A/cm^2)	10
CuNi-CeO_2 | **LSGM** | BSCF (ESC)	200	2.8	0.8 at 700°C<br>at 800°C	100<br>(0.58 A/cm^2)	11
PLSBSCF | **LSGM** | PLSBSCF (ESC)	230	3.7	0.3 at 700°C<br>1.2 at 800°C	220<br>(1.0 A/cm^2)	12
SFNM | LDC | **LSGM** | LSCF-SDC (ESC)	150	1.6	0.5 at 700°C<br>1.4 at 800°C	200<br>(0.5 A/cm^2)	13
CoNi@SDC | SDC | **LSGM** | SDC | LSCF-SDC (ESC)	145	1.5	1.69 at 800°C	2,000<br>(1.0 A/cm^2)	1
LSCF-SDC | **LSGM** | PBC (ESC)	200	2.8	0.95 at 800°C	50<br>(0.32 A/cm^2)	14
Ni-LCT | GDC | **ScCeSZ** | LSCF-GDC (ESC)	145	1.4	0.6 at 850°C	48<br>(0.6 A/cm^2)	15
LSCM-GDC | **ScCeSZ** | LSCF-GDC (ESC)	200	2.6	0.23 at 800°C	100<br>(0.3 A/cm^2)	16
SCFCO | **ScCeSZ** | LSM-GDC (ESC)	400	9.7	1.0 at 800°C	16<br>(1.0 A/cm^2)	17
SFM-GDC | GDC | **ScCeSZ** | GDC | SFM-GDC (ESC)	150	1.5	0.41 at 700°C<br>0.64 at 800°C	100<br>(0.37 A/cm^2)	18
**Ni-8YSZ** | Ni-8YSZ | 8YSZ | GDC | LSM (CSC)	300	2.4	0.41 at 700°C<br>0.64 at 800°C	100<br>(0.37 A/cm^2)	19
**Ni-8YSZ** | Ni-8YSZ | 8YSZ | LSCF-GDC (CSC)	450	5.2	0.5 at 700°C<br>1.0 at 800°C	-	20
**Ni-8YSZ** | Ni-8YSZ | 8YSZ | GDC | LSC (CSC)	400	4.1	0.25 at 600°C<br>0.35 at 700°C	-	21
**Ni-3YSZ** | Ni-8YSZ | 8YSZ | GDC | LSCF-GDC (CSC)	300	3.9	1.0 at 800°C	1,000<br>(1.0 A/cm^2)	22
**Ni-8YSZ** | Ni-8YSZ | ScSZ | GDC | LSCF-GDC (CSC)	400	4.1	1.35 at 800°C	-	23
**Ni-8YSZ** | Ni-8YSZ | ScSZ | GDC | LSCF-GDC (CSC)	500	6.4	0.4 at 800°C	100<br>(0.8 A/cm^2)	24
**Ni-ScSZ** | Ni-SDC | SDC | ScCeSZ | SDC | LSCF-SDC (HCSC)	280	7.3	0.36 at 600°C<br>1.1 at 700°C<br>2.5 at 800°C	1,000<br>(1.0 A/cm^2)	This work

The font for support layer is bold. LCN: $LaCo_{0.6}Ni_{0.4}O_{3-\delta}$; GDC: Gd_2O_3-doped CeO_2; NMC: $Ni(Mn_{1/3}Cr_{2/3})_2O_4$; LCFNb: $La_{0.9}Ca_{0.1}Fe_{0.9}Nb_{0.1}O_{3-\delta}$; LSM: $(La_{0.8}Sr_{0.2})_{0.95}MnO_{3-\delta}$; LSCM: $La_{0.75}Sr_{0.25}Cr_{0.5}Mn_{0.5}O_{3+\delta}$; BSCF: $Ba_{0.6}Sr_{0.4}Co_{0.2}Fe_{0.8}O_{3-\delta}$; PLSBSCF: $Pr_{1/6}La_{1/6}Sm_{1/6}Ba_{1/6}Sr_{1/6}Ca_{1/6}FeO_{3-\delta}$; SFNM: $Sr_2Fe_{1.3}Ni_{0.2}Mo_{0.5}O_6$; LDC: $La_{0.4}Ce_{0.6}O_{2-\delta}$; PBC: $PrBaCo_2O_{5+\delta}$; LCT: $La_{0.4}Ca_{0.4}TiO_3$; SCFCO: $Sm_{0.9}Ca_{0.1}Fe_{0.9}Cu_{0.1}O_{3-\delta}$; SFM: $Sr_2Fe_{1.5}Mo_{0.5}O_{6-\delta}$; LSC: $La_{0.6}Sr_{0.4}CoO_3$.

Table S2. Physical parameters of each layer for different cells.

Cell	Layer	Thickness (μm)	Mass (mg/cm^2)
**HCSC**	Anode	28.0±4.0	13.1±1.9
	Buffer	7.5±1.2	5.4±0.9
	Electrolyte	8.2±0.4	4.7±0.2
	Buffer	7.5±1.2	5.4±0.9
	Cathode	20.3±3.0	7.4±1.1
	Support	290±30	105.1±10.9
**MCSC**	Anode	28.0±4.0	13.1±1.9
	Buffer	7.5±1.2	5.4±0.9
	Electrolyte	8.1±0.4	4.6±0.3
	Cathode	22.2±4.0	8.0±1.4
	Support	290±30	105.1±10.9
**ESC**	Anode	28.0±4.0	13.1±1.9
	Buffer	6.5±1.1	4.7±0.8
	Electrolyte	192.0±20.6	109.6±11.8
	Cathode	21.0±2.0	7.6±0.7

Supporting References

1. Ma, W. *et al.* Encapsulated Co–Ni alloy boosts high-temperature CO_2 electroreduction. *Nature* **641**, 1156–1161 (2025).
2. Liu, W. N., Sun, X., Khaleel, M. A. & Qu, J. M. Global failure criteria for positive/electrolyte/negative structure of planar solid oxide fuel cell. *J. Power Sources* **192**, 486–493 (2009).
3. Ni, D.-W. *et al.* Influence of temperature and atmosphere on the strength and elastic modulus of solid oxide fuel cell anode supports. *J. Power Sources* **311**, 1–12 (2016).
4. Du, Y., Sammes, N. M., Tompsett, G. A. & Bowden, M. Comparison of mechanical and thermal properties of the tubular LSGM, CGO, and YSZ electrolytes. *ECS Proc. Vol.* **2001–16**, 311 (2001).
5. Li, T. *et al.* Robust anode-supported cells with fast oxygen release channels for efficient and stable CO_2 electrolysis at ultrahigh current densities. *Small* **17**, 2007211 (2021).
6. Duan, N. *et al.* Multi-functionalities enabled fivefold applications of $LaCo_{0.6}Ni_{0.4}O_{3-\delta}$ in intermediate temperature symmetrical solid oxide fuel/electrolysis cells. *Nano Energy* **77**, 105207 (2020).
7. Duan, N. *et al.* Exploring $Ni(Mn_{1/3}Cr_{2/3})_2O_4$ spinel-based electrodes for solid oxide cells. *J. Mater. Chem. A* **8**, 3988–3998 (2020).
8. Liu, L. *et al.* Composite ceramic cathode $La_{0.9}Ca_{0.1}Fe_{0.9}Nb_{0.1}O_{3-\delta}/Sc_{0.2}Zr_{0.8}O_{2-\delta}$ towards efficient carbon dioxide electrolysis in zirconia-based high temperature electrolyser. *Int. J. Hydrogen Energy* **42**, 14905–14915 (2017).
9. Qian, B. *et al.* Ca-doped $La_{0.75}Sr_{0.25}Cr_{0.5}Mn_{0.5}O_3$ cathode with enhanced CO_2 electrocatalytic performance for high-temperature solid oxide electrolysis cells. *Int. J. Hydrogen Energy* **46**, 33349–33359 (2021).
10. Abu Hajer, A., Velraj, S., Daramola, D. A. & Trembly, J. P. Carbon dioxide reduction in solid oxide electrolyzer cells using transition metals infiltrated into $Gd_{0.1}Ce_{0.9}O_{1.95}$ (GDC10) scaffolds. *J. Power Sources* **572**, 233040 (2023).
11. Wang, W. *et al.* Enhanced carbon dioxide electrolysis at redox manipulated interfaces. *Nat. Commun.* **10**, 1550 (2019).
12. Yang, M. *et al.* Robust cathode for efficient CO_2 electrolysis driven by entropy engineering in solid oxide electrolysis cells. *ACS Energy Lett.* **9**, 3818–3827 (2024).
13. Liao, Y. *et al.* Unraveling double-exchange effect coupling spin modulation of $Sr_2Fe_{1.5}Mo_{0.5}O_{6-\delta}$ electrocatalyst for solid oxide cells. *J. Am. Chem. Soc.* **147**, 21003–21013 (2025).
14. Liu, H. *et al.* Optimizing surface electronic structure by Ba dispersion for enhanced high-temperature oxygen evolution reaction activity. *J. Am. Chem. Soc.* **147**, 24451–24460 (2025).
15. Kyriakou, V. *et al.* Co-electrolysis of H_2O and CO_2 on exsolved Ni nanoparticles for efficient syngas generation at controllable H_2/CO ratios. *Appl. Catal. B Environ.* **258**, 117950 (2019).

16. Zhou, J. *et al.* Study of CO_2 and H_2O direct co-electrolysis in an electrolyte-supported solid oxide electrolysis cell by aqueous tape casting technique. *Int. J. Hydrogen Energy* **44**, 28939–28946 (2019).
17. Wang, S., Deng, S., Hao, Z., Hu, X. & Zheng, Y. Ca/Cu co-doped $SmFeO_3$ as a fuel electrode material for direct electrolysis of CO_2 in SOECs. *Fuel Cells* **20**, 682–689 (2020).
18. Pandiyan, A. *et al.* CO_2 conversion via coupled plasma-electrolysis process. *J. CO_2 Util.* **57**, 101904 (2022).
19. Ebbesen, S. D. & Mogensen, M. Electrolysis of carbon dioxide in solid oxide electrolysis cells. *J. Power Sources* **193**, 349–358 (2009).
20. Zhan, Z. & Zhao, L. Electrochemical reduction of CO_2 in solid oxide electrolysis cells. *J. Power Sources* **195**, 7250–7254 (2010).
21. Lo Faro, M., Zignani, S. C., Trocino, S., Antonucci, V. & Aricò, A. S. New insights on the co-electrolysis of CO_2 and H_2O through a solid oxide electrolyser operating at intermediate temperatures. *Electrochimi. Acta* **296**, 458–464 (2019).
22. Shang, Y. *et al.* 3D microstructural characterization of Ni/yttria-stabilized zirconia electrodes during long-term CO_2 electrolysis. *J. Mater. Chem. A* **11**, 12245–12257 (2023).
23. Mahmood, A., Bano, S., Yu, J. H. & Lee, K.-H. High-performance solid oxide electrolysis cell based on ScSZ/GDC (scandia-stabilized zirconia/gadolinium-doped ceria) bi-layered electrolyte and LSCF (lanthanum strontium cobalt ferrite) oxygen electrode. *Energy* **90**, 344–350 (2015).
24. Yu, S.-B. *et al.* Syngas production in high performing tubular solid oxide cells by using high-temperature H_2O/CO_2 co-electrolysis. *Chem. Eng. J.* **335**, 41–51 (2018).
